# Assistance to children with Autism Spectrum Disorder: perceptions of healthcare professionals in a Pediatric Emergency Room

**DOI:** 10.1590/1980-220X-REEUSP-2025-0350en

**Published:** 2026-01-30

**Authors:** Camila Lovato de Figueiredo, Neila Santini de Souza, Aline Cammarano Ribeiro, Fernanda Luisa Buboltz, Hellen Thamara Quoos Santos, Eliane Tatsch Neves

**Affiliations:** 1 Universidade Federal de Santa Maria, Departamento de Enfermagem, Santa Maria, RS, Brazil.

**Keywords:** Child Health, Pediatric Nursing, Autism Spectrum Disorder, Emergency Service, Hospital, Patient Care Team

## Abstract

**Objective:**

To understand the care provided to children with Autism Spectrum Disorder (ASD) in a Pediatric Emergency Room from the perspective of healthcare professionals.

**Method:**

Qualitative research grounded in the framework of comprehensive care. Narrative interviews were conducted with 17 professionals from the nursing and medical staff of the Pediatric Emergency Room from March to May 2024. The interviews were subjected to inductive thematic analysis. The project was approved by the Research Ethics Committee.

**Results:**

The study highlighted three categories: professionals’ perceptions of families facing stress and a lack of knowledge about ASD; weaknesses in care, marked by the absence of protocols, difficulties in coordination within Health Care Networks (*RAS*), structural limitations, and insufficient nursing staffing; and strategies adopted, such as family collaboration, environmental adaptations, and flexibility in rules.

**Conclusion:**

Caring for children with ASD presents structural, organizational, and educational challenges that compromise the comprehensiveness of care. It is suggested that investments be made in continuing education, infrastructure improvements, and person-centered care practices, in accordance with the principles of the Brazilian Public Health System (*SUS*).

## INTRODUCTION

Autism Spectrum Disorder (ASD) is characterized by core symptoms related to social communication, restricted and repetitive behaviors, as well as related comorbidities, including sensory processing issues, feeding problems, and challenging behaviors^(1)^.

The Centers for Disease Control and Prevention (CDCs), a US government agency, identified a prevalence of one child with autism for every 36 8-year-old children^(2)^. In Brazil, the 2022 Demographic Census found that 2.4 million people reported having received a diagnosis of ASD (1.2% of the Brazilian population)^(3)^.

Given the growing recognition of ASD, it becomes essential to discuss the structure of health services aimed at this population. With this in mind, the Health Care Network (*RAS*) is organized into interconnected points of care, including thematic networks focused on the main needs of the population. The Network for Attention to People with Disabilities is one of the pillars and establishes guidelines for priority care for people with disabilities, including those with ASD. However, its implementation still presents inequalities, which compromises the effectiveness of care, especially in urgent and emergency settings^(4)^.

Another axis of the RAS is the Emergency Care Network, which is organized based on the relationship among various health care services, such as the Mobile Emergency Care Service (*SAMU*), the Emergency Care Units (*UPA*), and the Hospital Component, which includes the Pediatric Emergency Room, the empirical field of this study, characterized as a Hospital Emergency Department of the RAS^(5)^.

The RAS are based on the comprehensiveness of care, as outlined in Law 8.080/90, which regulates the Brazilian Public Health System (*SUS*), and is a fundamental principle encompassing an organizational approach to healthcare. It prioritizes horizontal actions in healthcare and values a model centered on the person, their individual needs, and the promotion of humanized care^(6)^.

The National Policy for Comprehensive Child Health Care (*PNAISC*) also underscores the importance of comprehensive care as a structuring axis for promoting children’s health. The policy recognizes that child health is influenced by multiple interrelated factors, making it essential for health services to consider all dimensions of life in their care practices^(7)^.

That being said, a literature search was conducted in August 2023 in the databases *National Library of Medicine* (PubMed) and *Web of Science* (WoS), using the search strategy [*“Autism” AND “Child” AND “Emergency medical services”*], for the planning and substantiation of the research. In summing the results from the sources of evidence, 139 studies were found, of which 36 were aligned with the research topic and were, for the most part, international.

Based on the analysis of the literature found based on the perspective of the comprehensive approach proposed by Law 8.080/90 and the *PNAISC*, it is possible to recognize that, although comprehensive care is a fundamental principle for children’s health, and the need for staff training is highlighted by national and international studies^(8,9)^, the urgent and emergency environment presents challenges that require an approach adapted to the needs of ASD children.

The presence of sensory stimuli, such as sounds, lights, smells, and touch, in emergency room environments can be aversive for these children, given their hypersensitivity^(10)^, thus requiring appropriate management by professionals when dealing with crises of psychic disorder caused by these numerous stimuli^(11)^. It is also reported that professionals lack knowledge about the specifics of the disorder, including signs and symptoms, effective communication strategies, and appropriate ways to involve family members in the care process^(12)^.

Although guidelines exist for the care of this population, significant gaps still persist in the understanding and practice of care in emergency services. Therefore, it is essential to investigate the perception and approach of healthcare professionals regarding these issues in the context of the Emergency Room, aiming to identify strategies that improve care and promote more effective and sensitive service to the needs of ASD children and their families.

In this context, the nursing central role stands out, since this professional category maintains continuous contact with patients in the hospital environment. Nurses are strategically positioned to identify barriers to care and implement strategies that promote comprehensive care, sensitive to the sensory, behavioral, and communicational needs of ASD children in emergency settings^(13)^.

Given the above, the question arose: how is care provided to children with autism in a Pediatric Emergency Room, from the perspective of the healthcare professionals working there? The objective was to understand the care provided to children with Autism Spectrum Disorder in a Pediatric Emergency Room from the perspective of healthcare professionals.

It is expected that the knowledge gained from this research will support the improvement of care and contribute to comprehensive care for children with autism in Pediatric Emergency Departments.

## METHOD

### Design of Study

Qualitative research^(14)^ anchored in the framework of comprehensive care of the *SUS*, as observed in Law 8.080/90^(6)^ and in the *PNAISC* ^(7)^. For the preparation of this manuscript, the recommendations of the Consolidated Criteria for Reporting Qualitative research (COREQ)^(15)^ were followed.

### Local

The study was conducted in a Pediatric Emergency Room of a medium-sized, high-complexity University Hospital located in Rio Grande do Sul. The Pediatric Emergency Room structure provides seven beds, one of which is for isolation and six are designated for the Conventional Care Unit. In addition, the unit has an emergency room with a stretcher.

This service is the main access point for urgent and emergency pediatric care in the central-western region of the state, serving as a referral center for 45 municipalities. The service consists of pediatricians and a nursing team providing 24-hour care, in addition to medical residents and a multidisciplinary team. This multidisciplinary team is not exclusive to the sector, but professionals such as physiotherapists, social workers, and nutritionists may be assigned to meet the needs of children and their families.

The children received by this service are those referred by *SAMU* in cases of serious emergency; by specialized services, such as neuropediatrics; and those who are being monitored after premature birth. After admission to the Pediatric Emergency Room, the child’s flow may involve discharge after treatment and stabilization of symptoms, or admission to the service and subsequent referral to the Pediatric Inpatient Unit or Pediatric Intensive Care Unit, depending on the case severity.

A study conducted at the same Emergency Room revealed a total of 3,830 patient visits between 2019 and 2023^(8)^. These include emergencies such as trauma, major burns, and severe metabolic disorders, as well as outpatient issues related to gastrointestinal, neurological, hematological-oncological diagnoses, congenital malformations, cardiovascular and genitourinary conditions, care for external causes, surgical cases, and, primarily, care for exacerbations of respiratory diagnoses^(8)^.

### Population and Selection Criteria

The study population was defined by convenience sampling, a type of non-probabilistic sampling in which participants are selected based on ease of access and their potential to respond to the survey. The aim was to interview the permanent healthcare professionals at the Pediatric Emergency Room, including pediatricians, registered nurses (regardless of specialization), and nursing technicians. The fact that these are the mandatory components of the basic team of a Pediatric Emergency Room, according to the National Policy for Emergency Care^(16)^, was taken into consideration.

The inclusion criterion was being a healthcare professional from the aforementioned categories, working in the study setting. Professionals who were on any type of leave or vacation during the data collection period were excluded. The research was disseminated within the study setting, and professionals were randomly invited to participate during their work shifts.

### Data Collection

The data collection procedure took place in person between March and May 2024. The researcher was already familiar with the field of research due to practical classes and internships during her undergraduate Nursing degree. Therefore, she re-established contact with the service, at which point the research was presented to the professionals and, after inviting them to participate in the study, the objectives of the research were explained. Of the 20 professionals working in the Pediatric Emergency Room, comprising 12 from the nursing team (7 nurses and 6 nursing technicians) and 7 from the medical team, 6 nurses, 5 nursing technicians, and 6 doctors were interviewed.

The narrative interview was conducted using a pre-prepared script, and a pilot test was carried out with nurses and nursing technicians from the research group who work with children and adolescents. The script contained questions to characterize the interviewee and the question that triggered the narrative interview: “Tell me how you perceive the care provided to autistic children in the Pediatric Emergency Room”.

The field notes were taken after the interview and incorporated into the interpretive process, complementing the transcribed accounts. These records helped contextualize the statements and identify environmental elements that influenced the participants’ experience, contributing to the robustness of the analysis.

The interviews were conducted solely by the undergraduate nursing researcher with experience in the field and in research. Data collection took place in a reserved room at the service during the morning, afternoon, and evening periods, and was audio-recorded, with an average duration of 10 minutes.

The sufficiency of the data was defined by the criterion of theoretical saturation, understood as the point at which the information obtained in the interviews began to show recurrence and consistency, without adding new elements relevant to the objectives of the study. This process was monitored concurrently with the collection and preliminary analysis of the data. After the 17th interview, repetition of content and convergence of speeches were observed among the different participants and professional categories, indicating saturation and, consequently, the sufficiency of the empirical material. There were no withdrawals after acceptance to participate in the interview.

### Data Analysis and Treatment

The recorded interviews were transcribed in full, without being returned to the participants, and subjected to inductive-reflective thematic content analysis^(17)^, consisting of six phases: 1) Familiarization with the data; 2) Generation of initial codes; 3) Search for themes; 4) Review of identified themes; 5) Definition and naming of themes; 6) Production of the report.

Three categories of analysis were organized: The families of ASD children in the Pediatric Emergency Room from the perspective of healthcare professionals; Weaknesses related to the care of ASD children in the Pediatric Emergency Room from the perspective of healthcare professionals; and Strategies adopted by healthcare professionals in the healthcare of ASD children in the Pediatric Emergency Room.

### Ethical Aspects

The study followed all the legal guidelines and prerogatives established by the National Health Council and was approved by the Research Ethics Committee (*CEP*) of the institution, with opinion number 6.512.409/2023. All participants received clear and objective explanations about the nature of the research, its purposes, and guidelines for conducting the interviews. The Free Informed Consent Form (FICF) was provided, which was signed in two copies, with one given to the participant and the other kept by the researcher.

To maintain the participants’ anonymity, codes referring to the professionals’ occupation were used, with ENF being adopted for Nurse (*Enfermeiro* in Portuguese), TE (*Técnico em Enfermagem*) for Nursing Technician, and MED (*Médico*) for Doctor, in addition to numbering in ascending order, in the order in which the interview was conducted.

## RESULTS

Based on the participants’ characterization, it was evident that, of the 17 included, six were nurses, five were nursing technicians, and six were doctors, among whom there was a prevalence of women, 16 (94.12%) of the total.

The participants were between 27 and 56 years old, with an average age of 42.30 years, and the length of time in their respective professions ranged from 4 to 29 years, with an average of 16.41 years. The length of time spent working in the Pediatric Emergency Room ranged from 1 month to 23 years, with an average of 7.52 years.

With regard to Graduate or Post-Technical degrees, 100% (17) of the participants reported having them, with 8 specializations being in the field of practice of these professionals, such as Pediatrics and Urgency and Emergency.

The results obtained from the analysis of the data obtained in the interviews were synthesized into 3 thematic categories described below.

*Families of ASD children in the Pediatric Emergency Room from the perspective of healthcare professionals.*

The professionals reported their perceptions of the families of children with autism during hospitalizations in the service, the impact of leaving the child’s familiar environment, and the families’ lack of knowledge about ASD.

*Many families get very stressed by the difficult management of the patient, especially because every time it takes them out of their comfort zone, they become more difficult. There are some procedures that the family finds a little more restrictive for them to do. This, consequently, reinforces the patient’s stress. (MED-1)*

*Sometimes parents don’t have much knowledge, or aren’t very interested; it varies a lot. (TE-1)*

*The family is not always prepared; sometimes mothers arrive here with a suspected diagnosis, but the diagnosis is not always confirmed, and the specific care required for these children is not always known. So they have difficulties, they arrive here full of doubts, and here at the emergency room we don’t have much time to talk about that. (ENF-5)*

Participants mentioned the inclusion of family members in care during visits to the Pediatric Emergency Room and their role as facilitators in this process.

*We ask for a lot of help from the parents, because we can’t just start by positioning the thermometer, for example, when we’ll check the signs. I give it to the mother to put on the child. So we try to just supervise the mother, so that the children don’t get nervous and agitated. (TE-2)*

*We try to attend to the children while respecting their limitations. Sometimes, if a patient is not very cooperative, we try to get help from the parents to hold them so we can do a physical exam. We try to adapt so as not to generate more stress for the child and the family. (MED-4)*

In summary, participants observe, while assisting ASD children, the stress and lack of knowledge of caregivers regarding the disorder, and realize the importance of involving caregivers in the care process, seeking to understand from the caregiver what the child’s limits are.

*Weaknesses related to the care of ASD children in a Pediatric Emergency Room from the perspective of healthcare professionals.*

Regarding the limitations present in the context of the Pediatric Emergency Room, professionals cited some barriers related to the difficulty of managing these children, such as the stigma associated with injections and venipuncture, as well as the psychological disorganization caused by the hospital environment.

*Depending on how they [the children] are feeling, if they are experiencing pain or discomfort, they generally show more resistance to contact, especially since they already associate it with venipuncture. (ENF-1)*

*They arrive afraid of the healthcare team; the issue of injections already makes them apprehensive. (ENF-3)*

*They get very stressed being here in the hospital environment, more stressed than the others. (TE-3)*

Professionals reported factors related to access to specialized services, early diagnosis, and teamwork as limitations that exist in the service and in society:

*The whole problem is with the network. A mother arrived with an autistic child on a walk-in basis because the teacher told her to come here. The mother said that the teacher mentioned that there are two other classmates who have special needs, and the teacher wanted a diagnosis to request an assistant. We talked to her so that she could contact the primary care unit; the general practitioner could then refer her to a pediatrician or directly to a neurologist. But the evaluation with the neurologist is taking a year and a half here, and 6 months privately. So, we said that she could choose paying a private consultation, but that it would be important for her to maintain her connection with the hospital, because it’s difficult to get in later. I think there are a lot of children in need who can’t get to the service. (ENF-4)*

*We have the specialists, but they take a while and often don’t talk to the nursing staff; they talk to the medical team, and sometimes we don’t even know what course of action to take with these children. (ENF-6)*

The Pediatric Emergency Room infrastructure is also reported as an obstacle, since the inpatient area does not have a private environment beyond isolation, which makes it difficult to protect ASD children from the numerous stimuli inherent to the unit.

Furthermore, they cite the number of professionals, with a nursing team consisting of one nurse and one nursing technician per shift for five beds and attending to external demands, as another challenge.

*It’s difficult because we don’t have an office; when it’s full, we don’t even have anywhere to see patients. (MED-3)*

*We don’t have a very good physical space that would allow us to provide care without noise or excessive stimulation, because when they need to be in the main room there are many beds, so this disturbs the autistic children. (MED-6)*

*There are only two of us here [a nurse and a nursing technician], so in some procedures it’s quite difficult for us to be able to perform. If it’s a more aggressive autistic child, we have difficulty [...] sometimes we even need to ask for help from other colleagues in other departments. (TE-4)*

Finally, some professionals mentioned feeling worried, experiencing difficulties, and fear when caring for a child with ASD, indicating a need for training to support these children.

*We only have more difficulty with the aggressive ones; I myself am afraid they might hit me, because there are children who are bigger than me. (TE-5)*

*I think the team itself provides good care for the patients, but perhaps what could be improved is some training for us on how to deal with these children, because I myself, as a professional, have never had any training or anything like that. (ENF-5)*

These limitations hinder comprehensive care, given that the lack of a suitable environment that meets the specific needs of any population, as well as feelings of worry, fear, and insecurity, in addition to a lack of specific qualifications, ultimately hinder the provision of qualified and comprehensive care.

*Strategies adopted by healthcare professionals in the care of ASD children in a Pediatric Emergency Room.*

Some professionals shared their perceptions regarding the team’s performance during the care of an ASD child.

*Here in the emergency room, I’ve noticed that the staff is more familiar with the specific needs of these patients, so I believe that the care provided is adequate, as far as possible. (MED-1)*

*Generally speaking, I think I manage it well, and the team is also very collaborative about it. (ENF-3)*

Some participants reported perceiving that they themselves, or the team in general, do not have significant problems related to the care of ASD children and use some adopted strategies to facilitate assistance.

*I had a case of suspected appendicitis in an autistic child with a significant degree of dependence, and we sedated her before sending her for a CT scan to prevent the child from becoming agitated by the change in environment. (MED-1)*

*When a child is very aggressive or highly hypersensitive, we isolate them when possible, or here in these consultation rooms, to try to reduce stimulation and have fewer people passing by. (MED-5)*

*Here in the Emergency Room we have some rules: when we treat a child, only one caregiver is allowed, because we have few beds and the physical space is small; when we have an ASD patient, we allow more than one family member to enter if necessary. (MED-2)*

As a final topic discussed as a way to ensure comprehensive care for this population, the professionals mentioned means of accessing the service where they work.

*Many end up being followed up by a neurologist; these are children linked to specialized outpatient care here at the hospital who end up seeking help for another urgent care at our emergency room because they are already linked to the service here. (ENF-2)*

*We have children who are being followed by a pediatric neurologist and have the red stamp, so they end up seeking care here at the Pediatric Emergency Room for other needs, not just a medication adjustment, for example. (ENF-3)*

In the case of children who have been referred to the specialized service and who receive follow-up care after premature birth, they have a link with the hospital through what is called a “red stamp”. Knowing that the University Hospital’s Emergency Room does not accept walk-ins, the red stamp guarantees access for this group of children to the Pediatric Emergency Room.

## DISCUSSION

The findings of this research, by highlighting the role of the family, limitations, and opportunities in the care of children with ASD in a Pediatric Emergency Room, engage with the complexity of comprehensive care in the context of emergency situations. Data categorization allowed for a critical analysis of the structural, relational, and organizational conditions that may hinder the effective delivery of comprehensive and humanized care.

The active participation of the family in care was identified by the interviewees as a facilitating element in assisting ASD children. Strategies such as assisting in approaching the child during vital sign assessment and maintaining dialogue between professionals and family members were cited as fundamental to the success of the care provided. These practices reflect the principles of family-centered care, a model that has proven effective in building bonds and reducing caregiver stress^(18)^.

However, gaps in the operationalization of the family-centered healthcare model were highlighted as one of several significant limitations related to the care of ASD children in Pediatric Emergency Departments. According to participants in a study, there is difficulty in finding a suitable time to provide guidance regarding the flows of the care network and specific care, for example. This failure by the team to provide information to family members can intensify caregiver stress, a situation that, in turn, has direct repercussions on the child’s well-being and behavior, impacting the entire care process^(19)^.

The PNAISC underscores the need for bonds between professionals, children, and families as a condition for providing comprehensive and effective care^(4)^. However, professionals report that the short time children spend in the service is a significant obstacle to building these bonds. The literature corroborates this finding by showing that a logic focused on the immediate resolution of demands can limit the establishment of deeper and more meaningful therapeutic relationships^(20)^.

Another limitation cited by professionals was the inadequacy of the service’s infrastructure, evidenced by noisy environments, intense lighting, and shared space with several patients – factors that contradict ASD children’s sensory needs. This reality is observed in international studies that point to the hospital environment as a potentially disruptive space for these children, intensifying agitation, resistance to procedures, and the risk of coercive interventions, such as physical or pharmacological restraint^(10,11)^.

A review study conducted in a pediatric emergency department shows that restraint is only justifiable in extreme situations and that its frequent use may reflect failures in adopting preventive approaches and in the training of healthcare teams. Proper management requires specific knowledge about the child’s clinical condition and non-pharmacological strategies for emotional regulation, which are often absent in the healthcare teams’ daily practice^(21)^.

An analysis of the specific characteristics of the study setting as addressed by the study participants reveals the complexity of providing care to ASD children in the Pediatric Emergency Room, especially when related to the lack of individual beds and private treatment areas. A qualitative study conducted with parents of children with ASD and emergency service professionals points to inadequate physical spaces as a significant barrier to effective care, especially related to patients with specific neuropsychological needs, such as children with autism^(9)^.

Furthermore, the scarcity of human resources and the lack of specialized training on the disorder were widely cited by interviewees as hindering comprehensive care and contributing to feelings of worry, fear, and insecurity. Research conducted in the same setting as this study also highlighted difficulties in care related to the environment’s infrastructure and the reduced number of nursing staff per shift^(22)^. Parents and caregivers often point to the absence of adapted communication strategies as a factor that increases the stress of the emergency room experience^(9)^.

The difficulties reported by professionals in the care pathway, such as delays in referrals to specialized services and late initiation of follow-up, reveal a fragile scenario in access to and comprehensiveness of care for ASD children. These obstacles compromise not only early diagnosis and timely intervention, but also health promotion and rehabilitation, pillars of the comprehensive care advocated by the PNAISC^(7,23)^.

It is important to emphasize that many of the weaknesses identified in this study go beyond the individual performance of healthcare professionals and are related to management responsibilities at different levels of *SUS* ^(14)^. At the local level, the management of the Emergency Room plays a role in adapting the infrastructure, defining care protocols, and promoting ongoing training for the teams. From a broader perspective, municipal, state, and federal managers must ensure structural investments and the implementation of public policies that guarantee adequate working conditions and well-defined referral flows.

Thus, overcoming the barriers encountered depends on institutional decisions that integrate planning, financing, and monitoring, reinforcing the shared responsibility between professionals and managers in the effective implementation of comprehensive care, as advocated by the National Policy for Emergency Care^(14)^.

Finally, it is worth highlighting that networking involves constructs such as teamwork and interprofessional collaboration, to offer a unique care project complemented by all multidisciplinary areas. However, the accounts from the healthcare professionals participating in the study reveal that, in some cases, this multidisciplinary teamwork does not occur, leaving some information restricted to medical professionals. Such practices should be avoided, given that only a complete multidisciplinary team has the potential to provide all the necessary, unique, and complementary support tailored to the specific manifestations of the disorder in each child^(24)^.

Regarding strategies adopted by professionals for managing ASD children in the Pediatric Emergency Room setting, the use of tools such as social stories – short, individualized stories that explain a social situation and represent a light care technology – by healthcare professionals can help people with ASD in interpreting and understanding social situations, such as hospital admission or the performance of a procedure. This type of strategy can improve communication between the team and the child who does not have an intellectual disability, helping to improve understanding and reduce stress related to a hyper-stimulating environment, such as an emergency room^(25)^.

Another method used by professionals to improve care for this population is the flexibility of routines and the use of isolation beds to protect the child from the stimuli of the service. In a retrospective review study, the professionals interviewed reported adopting compensatory strategies, such as those cited by the professionals in this study, to mitigate the effects of excessive stimuli and provide a minimally welcoming environment. Creating a more tranquil environment to meet the needs of children with ASD has proven effective in helping professionals provide sensory care^(26)^.

In addition to local planning, comprehensive care for children with ASD requires coordination between different points in the healthcare network, from primary care to high complexity care. The Emergency Care Network, for example, is fundamental and must operate in an interconnected way with the other strategic axes of the *PNAISC*, including the one focused on children with disabilities. This collaboration aims to guarantee continuous and coordinated care, capable of responding to the unique needs of each child and family^(7)^.

Tools like the Sunflower Lanyard and the CIPTEA contribute to the recognition of hidden disabilities and priority access to health services^(27,28)^. However, its implementation and effectiveness still require greater uniformity across the country.

Within the context of Primary Care, the central role of medical and nursing professionals in the early detection of warning signs of ASD stands out, especially during well-child visits. The use of instruments such as the M-CHAT-R/F, recommended by the Brazilian Society of Pediatrics and included in the Child’s Health Record, allows for an initial screening of behaviors suggestive of the disorder in children aged 16 to 30 months^(29)^. This strategy is in line with Law No. 13.438/2017, which establishes guidelines for assessing risks to psychological development in childhood^(30)^. Nevertheless, the effectiveness of this surveillance depends on the training of professionals, the continuity of care, and coordination with other levels of the network.

Regarding access to the tertiary care service in question, the professionals mentioned the adoption of the “Red Stamp” system, an instrument included in the Child’s Health Record for individuals who receive follow-up care from specialists, such as neuropediatrics, or who were born prematurely at this hospital. This strategy is designed as a means of ensuring access for greater complexity to children in a service that does not operate on an open-door basis for spontaneous demands and, therefore, aligns with the principles of comprehensiveness and universality within the SUS^(6)^.

Thus, by recognizing the challenges and possibilities involved in providing care to children with Autism Spectrum Disorder in a Pediatric Emergency Room, this study reinforces the need for integrated and operational care networks, as well as qualified professionals at all levels of healthcare. It is important to emphasize that workforce qualification and coordination between points in the network are essential conditions to guarantee child- and family-centered care, as advocated by the *SU*S guidelines.

Regarding the limitations of the study, the average interview duration, approximately 10 minutes, can be justified by the context of the Pediatric Emergency Room, an environment characterized by intense demand for care and limited time for professionals. Despite this, the methodological rigor followed by the researcher and the targeted trigger question made it possible to obtain objective accounts focused on the phenomenon under investigation.

Finally, it is acknowledged that the protocol was tested only with nurses and nursing technicians from the authors’ research group, and did not include physicians. The evaluation of the guiding question was considered relevant due to the broad scope of the Nursing perspective in Emergency Room care. Furthermore, during data collection, no difficulties in understanding the question were reported by the other participants, which reinforces the suitability of the script for all professional profiles included.

## CONCLUSION

It was concluded that healthcare professionals in the Pediatric Emergency Room recognize both strengths and weaknesses in the care of children with ASD. Among the main findings, the perception of the impact of hospitalization on families stands out, often marked by stress and doubt, which reinforces the importance of including caregivers in the care process as mediators, while simultaneously providing health education.

Since the analysis was based on the professionals’ perspective, it is not possible to state whether the care provided is comprehensive or not, but evidence points to weaknesses, such as the absence of standardized protocols and difficulties in coordination with the RAS, structural limitations of the service – especially the lack of environments with less sensory stimulation – and the insufficient size of the nursing staff. Insecurities among professionals in clinical management were also reported, indicating a need for specific training and continuing health education, an essential strategy for expanding technical and interpersonal skills.

In contrast, adaptive strategies emerged, such as the relaxation of institutional rules, the use of the “Red Stamp” to guarantee access to the service, and the collaboration of family members, pointing to possible ways to improve the quality of care. In this regard, the importance of carrying out multiprofessional clinical meetings is highlighted as they are spaces for dialogue and the exchange of knowledge, which strengthens shared decision-making. Additionally, there is still a need for less prescriptive and more person-centered care practices, in which the uniqueness of the child and the family is recognized. This perspective, in line with the *SUS*’s principle of comprehensiveness, contributes to the humanization of care, the reduction of traumatic experiences, and the implementation of more embracing and effective care.

This study stands out for its originality in the Brazilian context, addressing the care of ASD children in Pediatric Emergency Rooms, making a significant contribution to the scientific literature on the subject and offering support for future reflections and a theoretical framework for management to use in creating protocols and new service flows.

Finally, this study implications for practice contribute both to nursing, which needs to equip itself and develop qualified reception strategies, and to the management of services, which must invest in structural conditions, adequate staffing, and training policies, thus ensuring care based on comprehensiveness.

## DATA AVAILABILITY

The entire dataset supporting the results of this study was published in the article itself.

## References

[1] American Psychiatric Association (2022). Diagnostic and statistical manual of mental disorders: DSM-5-TR. 5th
ed..

[2] Maenner MJ, Warren Z, Williams AR, Amaokahene E, Bakin AV, Bilder DA (2023). Prevalence and characteristics of autism spectrum disorder among
children aged 8 years: autism and developmental disabilities monitoring
network, 11 sites, United States, 2018.. MMWR Surveill Summ.

[3] Instituto Brasileiro de Geografia e Estatistica. (2025). Censo 2022 identifica 2,4 milhões de pessoas diagnosticadas com autismo
no Brasil..

[4] Brasil. (2012). Portaria nº 793 de 24 de abril de 2012. Institui a Rede de Cuidados à
Pessoa com Deficiência no âmbito do Sistema Único de Saúde..

[5] Brasil. (2011). Portaria nº 1.600 de 7 de julho de 2011. Reformula a Política Nacional
de Atenção às Urgências e institui a Rede de Atenção às Urgências no Sistema
Único de Saúde (SUS)..

[6] Brasil. (2021). Lei nº 8.080 de 19 de setembro de 1990. Dispõe sobre as condições para a
promoção, proteção e recuperação da saúde, a organização e o funcionamento
dos serviços correspondentes e dá outras providências..

[7] Brasil. (2018). Ministério da Saúde. Política Nacional de Atenção Integral à Saúde da
Criança: orientações para implementação..

[8] Figueiredo CL, Ribeiro AC, Buboltz FL, Souza NS, Santos HTQ, Neves ET (2024). Caracterização de crianças com Transtorno do Espectro Autista
atendidas em Pronto-Socorro Pediátrico.. Rev Soc Bras Enferm Pedriatr..

[9] Nicholas DB, Muskat B, Zwaigenbaum L, Greenblatt A, Ratnapalan S, Kilmer C (2020). Patient- and Family - Centered Care in the Emergency Department
for Children With Autism.. Pediatrics.

[10] Wood EB, Halverson A, Harrison G, Rosenkranz A (2019). Creating a sensory-friendly pediatric emergency
department.. J Emerg Nurs.

[11] McGonigle JJ, Venkat A, Beresford C, Campbell TP, Gabriels RL (2014). Management of agitation in individuals with autism spectrum
disorders in the emergency department.. Child Adolesc Psychiatr Clin N Am.

[12] Garrick A, Lee ML, Scarffe C, Attwood T, Furley K, Bellgrove MA (2022). An Australian cross-sectional survey of parents’ experiences of
emergency department visits among children with autism spectrum
disorder.. J Autism Dev Disord.

[13] Mahoney WJ, Villacrusis M, Sompolski M, Iwanski B, Charman A, Hammond C (2021). Nursing care for pediatric patients with autism spectrum
disorders: A cross-sectional survey of perceptions and
strategies.. J Spec Pediatr Nurs.

[14] Minayo MCS (2008). O desafio do conhecimento..

[15] Souza VRS, Marziale MHP, Silva GTR, Nascimento PL (2021). Tradução e validação para a língua portuguesa e avaliação do guia
COREQ.. Acta Paul Enferm.

[16] Brasil. (2003). Ministério da Saúde. Política Nacional de Atenção às Urgências..

[17] Braun V, Clarke V (2019). Reflecting on reflexive thematic analysis.. Qual Res Sport Exerc Health.

[18] Snow SL, Smith IM, Latimer M, Cameron ES, Fox J, Chorney J (2022). A balancing act: an interpretive description of healthcare
providers’ and families’ perspective on the surgical experiences of children
with autism spectrum disorder.. Autism.

[19] Rochat CP, Gaucher N, Bailey B (2018). Measuring anxiety in the pediatric emergency
department.. Pediatr Emerg Care.

[20] Cavalli GC, Mosquéra JM, Ramos LF, Alves AR, Hanna MD, Napoli AE (2021). Relação entre a qualidade das prescrições médicas e a compreensão
do paciente: uma revisão de literatura.. Braz J Health Rev..

[21] Foster AA, Saidinejad M, Li J (2024). Approach to acute agitation in the pediatric emergency
department.. Curr Opin Pediatr.

[22] Silva JH, Buboltz FL, Silveira A, Neves ET, Portela LJ, Jantsch LB (2017). Permanência de familiares no atendimento de emergência
pediátrica: percepções da equipe de saúde.. Rev Baiana Enferm.

[23] Singhi P, Malhi P (2023). Early diagnosis of autism spectrum disorder: what the
pediatricians should know.. Indian J Pediatr.

[24] Costa NM, Santos PR, Beluco AC (2021). A importância da equipe multiprofissional de crianças diagnosticadas com
TEA. In: Almeida FA, organizador. Autismo: avanços e desafios..

[25] Pettersson E, Christensen BM, Berglund IG, Nylander E, Huus K (2024). Children with autism spectrum disorder in high technology
medicine environments; a qualitative systematic review of parental
perspectives.. Syst Rev.

[26] Bourke EM, Say DF, Carison A, Hill A, Craig S, Hiscock H (2021). Emergency mental health presentations in children with autism
spectrum disorder and attention deficit hyperactivity
disorder.. J Paediatr Child Health.

[27] Brasil. (2021). Lei nº 14.624 de 17 de julho de 2023. Altera a Lei nº 13.146, de 6 de
julho de 2015 (Estatuto da Pessoa com Deficiência), para instituir o uso do
cordão de fita com desenhos de girassóis para a identificação de pessoas com
deficiências ocultas..

[28] Brasil. (2020). Lei nº 13.977 de 8 de janeiro de 2020. Altera a Lei nº 12.764, de 27 de
dezembro de 2012 (Lei Berenice Piana), e a Lei nº 9.265, de 12 de fevereiro
de 1996, para instituir a Carteira de Identificação da Pessoa com Transtorno
do Espectro Autista (Ciptea), e dá outras providências..

[29] Brasil. (2017). Lei nº 13.438, de 26 de abril de 2017. Altera a Lei nº 8.069, de 13 de
julho de 1990 (Estatuto da Criança e do Adolescente), para tornar
obrigatória a adoção pelo Sistema Único de Saúde (SUS) de protocolo que
estabeleça padrões para a avaliação de riscos para o desenvolvimento
psíquico das crianças..

[30] Sociedade Brasileira de Pediatria (2017). Triagem precoce para Autismo/Transtorno do Espectro Autista..

