# Supplementary material for: Assistance to children with Autism Spectrum Disorder: perceptions of healthcare professionals in a Pediatric Emergency Room
Source: Rev Esc Enferm USP. 2026 Jan 30;60:e20250350. doi: 10.1590/1980-220X-REEUSP-2025-0350en (PMC12857795; doi:10.1590/1980-220X-REEUSP-2025-0350en)
Supplement: Translation

# Translation: Assistência às crianças com Transtorno do Espectro Autista: percepção dos profissionais de saúde em Pronto-Socorro Pediátrico

**DOI:** 10.1590/1980-220X-REEUSP-2025-0350pt

**Authors:** Camila Lovato de Figueiredo, Neila Santini de Souza, Aline Cammarano Ribeiro, Fernanda Luisa Buboltz, Hellen Thamara Quoos Santos, Eliane Tatsch Neves

**Affiliations:** 1 Universidade Federal de Santa Maria, Departamento de Enfermagem, Santa Maria, RS, Brasil.

**Keywords:** Saúde da Criança, Enfermagem Pediátrica, Transtorno do Espectro Autista, Unidades de Emergência, Equipe de Assistência ao Paciente

## Abstract

**Objetivo:**

Conhecer a assistência às crianças com Transtorno do Espectro Autista (TEA) atendidas em Pronto-Socorro Pediátrico a partir da perspectiva dos profissionais de saúde.

**Método:**

Pesquisa qualitativa ancorada no referencial da integralidade do cuidado. Desenvolveram-se entrevistas narrativas com 17 profissionais da equipe de enfermagem e médica do Pronto-Socorro Pediátrico de março a maio de 2024. As entrevistas foram submetidas à análise temática indutiva. O projeto foi aprovado pelo Comitê de Ética em Pesquisa.

**Resultados:**

O estudo evidenciou três categorias: a percepção dos profissionais sobre as famílias, que enfrentam estresse e desconhecimento sobre o TEA; as fragilidades do atendimento, marcadas pela ausência de protocolos, dificuldades de articulação nas Redes de Atenção à Saúde (RAS), limitações estruturais e de dimensionamento da equipe de enfermagem; e as estratégias adotadas, como a colaboração dos familiares, adaptações no ambiente e flexibilização de normas.

**Conclusão:**

O cuidado às crianças com TEA apresenta desafios estruturais, organizacionais e formativos, que comprometem a integralidade da assistência. Sugere-se investir em educação permanente, adequação da infraestrutura e práticas de cuidado centradas na pessoa, em conformidade com os princípios do SUS.

## INTRODUÇÃO

O Transtorno do Espectro Autista (TEA) caracteriza-se por sintomas centrais, relacionados à comunicação social, comportamentos restritos e repetitivos, além de comorbidades relacionadas, incluindo condições sensoriais, problemas de alimentação e comportamentos desafiadores^(1)^.

O *Centers for Disease Control and Prevention* (CDC), órgão governamental dos Estados Unidos, identificou a prevalência de uma criança com autismo a cada 36 crianças de 8 anos de idade^(2)^. No Brasil, o Censo Demográfico de 2022 identificou que 2,4 milhões de pessoas declararam ter recebido o diagnóstico de TEA (1,2% da população brasileira)^(3).^

Diante do crescente reconhecimento do TEA, torna-se essencial discutir a estrutura dos serviços de saúde voltados a essa população. Tendo isso em vista, a Rede de Atenção à Saúde (RAS) organiza-se em pontos de atenção articulados, incluindo redes temáticas voltadas às principais demandas da população. A Rede de Atenção à Pessoa com Deficiência configura-se como um dos eixos e estabelece diretrizes para o atendimento prioritário a pessoas com deficiência, incluindo pessoas com TEA. No entanto, sua implementação ainda apresenta desigualdades, o que compromete a efetividade da assistência, especialmente em ambientes de urgência e emergência^(4)^.

Outro eixo da RAS é a Rede de Atenção às Urgências, que se organiza a partir da relação entre diversos serviços de atenção à saúde, como o Serviço de Atendimento Móvel de Urgência (SAMU), as Unidades de Pronto Atendimento (UPA) e o Componente Hospitalar, onde se insere o Pronto-Socorro Pediátrico, campo empírico desse estudo, caracterizado como uma Porta Hospitalar de Urgência da RAS^(5)^.

As RAS são pautadas na integralidade do cuidado, presente na Lei 8.080/90, que regulamenta o Sistema Único de Saúde (SUS) e é um princípio fundamental que compreende uma abordagem organizacional da assistência. Esta prioriza a horizontalidade das ações em saúde e valoriza um modelo centrado na pessoa, em suas demandas individuais e na promoção da humanização do atendimento^(6)^.

A Política Nacional de Atenção Integral à Saúde da Criança (PNAISC) também reforça a importância da integralidade do cuidado como eixo estruturante para a promoção da saúde infantil. A política reconhece que a saúde da criança é influenciada por múltiplos fatores inter-relacionados, sendo fundamental que serviços de saúde considerem todas as dimensões da vida em suas práticas assistenciais^(7)^.

Isso posto, procedeu-se uma pesquisa bibliográfica no mês de agosto de 2023 nas bases de dados *National Library of Medicine* (PubMed) e *Web of Science* (WoS), utilizando a estratégia de busca [*“Autism” AND “Child” AND “Emergency medical services”*], para o planejamento e fundamentação da pesquisa. Na soma dos resultados das fontes de evidência, foram identificados 139 estudos, dos quais 36 estavam alinhados com o tema da pesquisa e eram, na sua grande maioria, internacionais.

A partir da análise da literatura encontrada pela ótica da integralidade proposta pela Lei 8.080/90 e pela PNAISC, é possível reconhecer que, embora a integralidade do cuidado seja um princípio fundamental para a saúde infantil e a necessidade de preparo da equipe seja evidenciada por estudos nacionais e internacionais^(8,9)^, o ambiente de urgência e emergência apresenta desafios que exigem uma abordagem adaptada às necessidades das crianças com TEA.

A presença de estímulos sensoriais, como sons, luzes, cheiros e toques, em ambientes de Pronto-Socorro, pode ser aversiva para essas crianças, dada sua hipersensibilidade^(10)^, sendo necessário o manejo adequado dos profissionais frente a crises de desordem psíquica causadas por esses inúmeros estímulos^(11)^. É relatado, ainda, o déficit de conhecimento dos profissionais sobre as especificidades do transtorno, incluindo sinais e sintomas, estratégias de comunicação eficazes e formas adequadas de envolver os familiares no processo de cuidado^(12)^.

Embora existam diretrizes para a atenção a essa população, ainda persistem lacunas significativas na compreensão e na prática do cuidado em serviços de emergência. Assim, é fundamental investigar a percepção e a abordagem dos profissionais de saúde em relação a essas questões no contexto do Pronto-Socorro, visando identificar estratégias que melhorem a assistência e promovam um atendimento mais eficaz e sensível às necessidades das crianças com TEA e suas famílias.

Nesse contexto, destaca-se o papel central da enfermagem, uma vez que essa categoria profissional mantém contato contínuo com os pacientes no ambiente hospitalar. Enfermeiros estão em posição estratégica para identificar barreiras no cuidado e implementar estratégias que favoreçam um atendimento integral, sensível às demandas sensoriais, comportamentais e comunicacionais de crianças com TEA em contextos de emergência^(13)^.

Diante do exposto, questionou-se: como ocorre a assistência às crianças com autismo em um Pronto-Socorro Pediátrico a partir da concepção dos profissionais de saúde que ali atuam? E objetivou-se conhecer a assistência às crianças com Transtorno do Espectro Autista atendidas em Pronto-Socorro Pediátrico a partir da perspectiva dos profissionais de saúde.

Espera-se que o conhecimento obtido a partir dessa investigação subsidie a qualificação do cuidado e contribua para um atendimento integral à criança com autismo em serviços de Pronto-Socorro Pediátrico.

## MÉTODO

### Tipo de Estudo

Pesquisa qualitativa^(14)^ ancorada no referencial da integralidade do cuidado do SUS, observado na Lei 8.080/90^(6)^ e na PNAISC^(7)^. Para a elaboração deste manuscrito foram seguidas as recomendações do *Consolidated criteria for reporting qualitative research* (COREQ)^(15)^.

### Cenário do Estudo

O estudo foi desenvolvido em um Pronto-Socorro Pediátrico (Ps Ped) de um Hospital Universitário de médio porte e alta complexidade, localizado no Rio Grande do Sul. A estrutura do PS Ped conta com sete leitos, sendo um de isolamento e seis destinados à Unidade de Cuidado Convencional. Além disso, a unidade possui uma sala de emergência com uma maca.

Esse serviço é a principal via de acesso para a assistência pediátrica de urgência e emergência da região Centro-Oeste do estado, sendo referência para 45 municípios. O serviço é composto por médicos pediatras e equipe de enfermagem que fornecem atendimento 24 horas, além de residentes médicos e equipe multiprofissional, a qual não é exclusiva do setor, mas profissionais fisioterapeutas, assistentes sociais e nutricionistas, por exemplo, podem ser alocados a fim de atender as demandas das crianças e suas famílias.

As crianças recebidas por esse serviço são aquelas encaminhadas pelo SAMU em caso de emergência grave; por serviço especializado, como a neuropediatria; e crianças que mantêm acompanhamento após nascimento prematuro. Após entrada no PS Ped, o fluxo dessa criança pode ocorrer com a alta após tratamento e estabilização dos sintomas, ou internação no serviço e posterior encaminhamento para a Unidade de Internação Pediátrica ou Unidade de Terapia Intensiva Pediátrica, conforme a gravidade do caso.

Um estudo desenvolvido no mesmo Pronto-Socorro evidenciou o quantitativo de 3.830 atendimentos realizados entre 2019 e 2023^(8)^. Nestes, incluem-se emergências como traumas, grandes queimados e alterações metabólicas graves, além de questões ambulatoriais relacionadas a diagnósticos gastrointestinais, neurológicos, hemato-oncológicos, malformações congênitas, cardiovasculares e geniturinários, assistência a causas externas, casos cirúrgicos e, principalmente, assistência a exacerbações de diagnósticos respiratórios^(8)^.

### Participantes e Critérios de Seleção

A população da pesquisa foi definida por conveniência, sendo um tipo de amostragem não probabilística em que os participantes são selecionados com base na facilidade de acesso e no seu potencial de responder a pesquisa. Buscou-se entrevistar os profissionais de saúde fixos do Pronto-Socorro Pediátrico, sendo eles médicos especialistas em pediatria, enfermeiros assistenciais, independente de especialização, e técnicos de enfermagem. Levou-se em consideração que esses são os componentes obrigatórios da equipe básica de um Pronto-Socorro Pediátrico, conforme a Política Nacional de Atenção às Urgências^(16)^.

O critério de inclusão foi ser profissional de saúde das categorias citadas, atuante no cenário do estudo. Foram excluídos os profissionais que estiveram em qualquer tipo de licença ou férias no período de coleta de dados. A pesquisa foi divulgada no cenário do estudo e os profissionais foram sendo convidados a participar da pesquisa de forma aleatória durante os turnos de trabalho.

### Coleta de Dados

O procedimento de coleta de dados ocorreu, presencialmente, no período de março a maio de 2024. A pesquisadora já possuía familiarização com o campo de pesquisa devido à realização de aulas práticas e estágios da Graduação em Enfermagem. Sendo assim, realizou a reaproximação com o serviço, momento em que a pesquisa foi apresentada aos profissionais e, após, realizado o convite para participação no estudo, esclareceu-se os objetivos da pesquisa. Dos 20 profissionais atuantes no PS Ped, sendo 12 da equipe de enfermagem (7 enfermeiros e 6 técnicos de enfermagem) e 7 da equipe médica, foram entrevistados 6 enfermeiros, 5 técnicos de enfermagem e 6 médicos.

A entrevista narrativa foi realizada com um roteiro previamente elaborado e um teste piloto foi realizado com enfermeiros e técnicos de enfermagem do grupo de pesquisa que trabalham com crianças e adolescentes. O roteiro continha perguntas para a caracterização do entrevistado e a pergunta disparadora da entrevista narrativa: “Fale-me como você percebe o atendimento às crianças autistas no Pronto-Socorro Pediátrico?”.

As notas de campo foram feitas após a entrevista e incorporadas ao processo interpretativo, complementando os relatos transcritos. Esses registros auxiliaram na contextualização das falas e na identificação de elementos do ambiente que influenciaram a experiência dos participantes, contribuindo para a robustez da análise.

As entrevistas foram realizadas somente pela pesquisadora graduanda de Enfermagem com experiência no cenário e em pesquisa. As coletas de dados ocorreram em uma sala reservada do serviço nos períodos da manhã, tarde e noite, e audiogravadas, tendo duração média de 10 minutos.

A suficiência dos dados foi definida pelo critério de saturação teórica, entendido como o ponto em que as informações obtidas nas entrevistas passaram a apresentar recorrência e consistência, sem acrescentar novos elementos relevantes para os objetivos do estudo. Esse processo foi monitorado de forma concomitante à coleta e à análise preliminar dos dados. Após a 17ª entrevista, observou-se repetição dos conteúdos e convergência das falas entre os diferentes participantes e categorias profissionais, o que indicou a saturação e, consequentemente, a suficiência do material empírico. Não houve desistências após o aceite em participar da entrevista e nem recusas.

### Análise e Tratamento dos Dados

As entrevistas gravadas foram transcritas na íntegra, sem terem sido devolvidas aos participantes, e submetidas à análise de conteúdo Temática Indutiva Reflexiva^(17)^, constituída por seis fases: 1) Familiarização com seus dados; 2) Geração de códigos iniciais; 3) Busca por temas; 4) Revisão dos temas identificados; 5) Definição e nomeação temas; 6) Produção do relatório.

Três categorias de análise foram organizadas: A família de crianças com TEA em Pronto-Socorro Pediátrico pela perspectiva dos profissionais de saúde; Fragilidades relacionadas ao cuidado de crianças com TEA em Pronto Socorro Pediátrico na perspectiva dos profissionais de saúde; e Estratégias adotadas pelos profissionais de saúde na atenção à saúde de crianças com TEA em Pronto-Socorro Pediátrico.

### Aspectos Éticos

O estudo seguiu todas as diretrizes e prerrogativas legais estabelecidas pelo Conselho Nacional de Saúde e foi aprovado pelo Comitê de Ética em Pesquisa (CEP) da instituição, sob o número de parecer 6.512.409/2023. Todos os participantes receberam explicações claras e objetivas sobre a natureza da pesquisa, seus propósitos e as orientações para a realização das entrevistas. Foi entregue o Termo de Consentimento Livre e Esclarecido (TCLE), o qual foi assinado em duas vias, sendo uma entregue ao participante e a outra mantida com a pesquisadora.

A fim de manter o anonimato dos participantes, utilizaram- se códigos referentes à profissão dos profissionais, adotando ENF para Enfermeiro, TE para Técnico de Enfermagem e MED para Médico, além de numeração em ordem crescente, na ordem em que a entrevista foi realizada.

## RESULTADOS

A partir da caracterização dos participantes, foi evidenciado que, dos 17 incluídos, seis eram enfermeiros, cinco técnicos de enfermagem e seis médicos, entre os quais houve prevalência do sexo feminino, com 16 (94,12%) do total.

Os participantes possuíam entre 27 e 56 anos de idade, com média de 42,30 anos, e o tempo de formação em suas respectivas profissões variou de 4 a 29 anos, com média de 16,41 anos. Já o tempo de atuação no Pronto-Socorro Pediátrico variou de 1 mês a 23 anos, com média de 7,52 anos.

No que diz respeito à Pós-Graduação ou Pós-Técnico, 100% (17) dos participantes referiram possuir, sendo que em 8 dos casos as especializações eram na área da atuação destes profissionais, como Pediatria e Urgência e Emergência.

Os resultados obtidos a partir da análise dos dados obtidos nas entrevistas foram sintetizados em 3 categorias temáticas descritas a seguir.

*A família de crianças com TEA em Pronto-Socorro Pediátrico pela perspectiva dos profissionais de saúde*

Os profissionais relataram a sua percepção acerca das famílias de crianças com autismo durante as internações no serviço, o impacto de sair do ambiente em que a criança está habituada e a falta de conhecimento das famílias sobre o TEA.

*Muitas famílias ficam bem estressadas com a situação de dificuldade de manejo do paciente, especialmente porque toda vez que sai do contexto deles, eles ficam mais difíceis. Alguns procedimentos a família fica um pouco mais impeditiva para fazer. Isso, consequentemente, retroalimenta o estresse do paciente. (MED-1)*

*Às vezes os pais não têm muito conhecimento, ou não tem muito interesse, isso varia muito. (TE-1)*

*A família nem sempre está preparada, às vezes as mães chegam aqui com uma suspeita de diagnóstico, nem sempre já tem o diagnóstico formado e se sabe os cuidados diferenciados para essas crianças. Então eles têm dificuldades, chegam aqui cheios de dúvidas e aqui no PS não é um lugar que a gente tenha muito tempo de conversar sobre isso. (ENF-5)*

Os participantes referiram a inclusão do familiar no cuidado durante o atendimento no PS Ped e a atuação dele como facilitador neste processo.

*A gente pede muito auxílio dos pais, porque a gente não chega já colocando o termômetro, por exemplo, quando a gente vai ver sinais. Eu dou pra mãe colocar na criança. Então a gente procura ficar mais só supervisionando a mãe, até para eles não ficarem nervosos e agitados. (TE-2)*

*A gente tenta atender as crianças respeitando as limitações que eles têm, às vezes se é um paciente pouco colaborativo, a gente tenta a ajuda dos pais para pegar no colo para a gente fazer um exame físico, a gente tenta adaptar para não gerar um stress maior para a criança e para a família. (MED-4)*

Em suma, os participantes observam, durante a assistência a crianças com TEA, o estresse e desconhecimento dos cuidadores acerca do transtorno e percebem a importância de vincular os responsáveis no cuidado, buscando ouvir do acompanhante quais são os limites da criança.

*Fragilidades relacionadas ao cuidado de crianças com TEA em Pronto Socorro Pediátrico na perspectiva dos profissionais de saúde*

Acerca das limitações presentes no contexto do Pronto-Socorro Pediátrico, os profissionais citaram algumas barreiras que se relacionam com a dificuldade de manejo dessas crianças, como o estigma associado a injeções e punção venosa, além da desorganização psíquica causada pelo ambiente hospitalar.

*Dependendo de como eles estão [as crianças], se estão sentindo dor, desconforto, aí geralmente apresentam mais resistência ao contato, até porque eles já relacionam com a questão da punção venosa. (ENF-1)*

*Eles chegam com medo da equipe de saúde, pela questão de injeções eles já ficam apreensivos. (ENF-3)*

*Eles ficam bem estressados de estar aqui no ambiente hospitalar, mais estressados que os outros. (TE-3)*

Os profissionais relataram fatores relacionados ao acesso a serviços especializados, ao diagnóstico precoce e o trabalho em equipe como limitações que existem no serviço e na sociedade:

*A falha toda é na rede. Chegou uma mãe com uma criança autista em uma demanda espontânea porque a professora disse que era pra vir aqui. A mãe disse que a professora falou que tem dois outros coleguinhas que são especiais, a professora queria ter um diagnóstico para conseguir pedir um monitor. A gente conversou com ela para que entrasse em contato com a unidade básica, o clínico poderia fazer a solicitação para um pediatra ou direto para um neurologista. Só que a avaliação com o neuro está demorando 1 ano e meio aqui e particular 6 meses. Então, a gente falou que poderia optar por fazer particular, mas que seria importante ela manter o vínculo aqui com o hospital, porque depois para entrar é difícil. Penso que tem um monte de criança precisando, que não consegue chegar ao serviço. (ENF-4)*

*A gente tem os especialistas, só que eles demoram um pouco e, muitas vezes, não falam com a equipe de enfermagem, eles falam com a equipe médica e, às vezes, a gente não sabe nem que conduta adotar com essas crianças. (ENF-6)*

A infraestrutura do Pronto-Socorro Pediátrico também é relatada como um obstáculo, já que a área de internação não possui um ambiente privativo além do isolamento, o que dificulta a proteção de crianças com TEA a inúmeros estímulos que são inerentes à unidade.

Além disso, citam o quantitativo profissional, com uma equipe de enfermagem constituída por um enfermeiro(a) e uma técnica de enfermagem por turno para cinco leitos e atendimento às demandas externas, como outro desafio.

*É difícil porque a gente não tem consultório, quando está cheio a gente não tem nem onde atender. (MED-3)*

*A gente não tem um espaço físico muito bom que proporcione com que a gente consiga fazer um atendimento sem barulho, sem muito estímulos, porque quando elas precisam ficar no salão tem muitos leitos, então isso perturba as crianças autistas. (MED-6)*

*Nós somos só duas aqui [uma enfermeira e uma técnica de enfermagem], então em alguns procedimentos é bem difícil a gente conseguir atuar. Se é uma criança autista mais agressiva a gente tem dificuldade [...] às vezes a gente precisa até pedir ajuda para outros colegas de outros setores. (TE-4)*

Por fim, alguns profissionais citaram que se sentem preocupados, com dificuldades e medo ao prestar cuidado a uma criança com TEA, sinalizando a necessidade de treinamento para acompanhar essas crianças.

*Somente os agressivos a gente tem mais dificuldade, eu mesma tenho medo de eles baterem, porque tem crianças que são maiores que eu. (TE-5)*

*A equipe em si eu acho que dá um atendimento bom para os pacientes, talvez o que poderia ter um pouquinho mais é um treinamento para a gente sobre como lidar com essas crianças, porque eu mesma enquanto profissional nunca tive uma capacitação ou alguma coisa assim. (ENF-5)*

Estas limitações dificultam um cuidado integral, tendo em vista que a falta de um ambiente adequado e que supra as necessidades específicas de qualquer população, assim como sentimentos de preocupação, medo e insegurança, além da falta de qualificação específica, acabam dificultando a realização de um cuidado qualificado e integral.

*Estratégias adotadas pelos profissionais de saúde na atenção à saúde de crianças com TEA em Pronto-Socorro Pediátrico*

Alguns profissionais relataram suas percepções acerca da atuação da equipe durante a assistência a uma criança com TEA.

*Aqui no pronto-socorro eu percebo que a equipe é mais habituada com as particularidades destes pacientes, então eu acredito que a assistência, dentro do possível, é adequada. (MED-1)*

*De uma maneira geral eu acho que eu consigo manejar bem e a equipe também é bem colaborativa quanto a isso. (ENF-3)*

Alguns participantes referiram perceber que eles próprios, ou a equipe no geral, não possuem problemas significativos relacionados ao cuidado de crianças com TEA e utilizam algumas estratégias adotadas para facilitar a assistência.

*Eu tive um caso de suspeita de apendicite em uma criança autista com grau de dependência importante e nós fizemos a sedação antes de encaminhar ela para a tomografia, para evitar que a criança ficasse agitada com a troca do ambiente. (MED-1)*

*Quando a criança é muito agressiva ou tem muita hipersensibilidade a gente coloca ela no isolamento quando é possível, ou aqui nesses leitos de consultório, para tentar deixar com um pouco menos de estímulo, com menos pessoas passando. (MED-5)*

*Aqui no Pronto-Socorro tem algumas regras: quando a gente atende uma criança só pode entrar um acompanhante, porque a gente tem poucos leitos e o espaço físico é pequeno, quando a gente tem um paciente com TEA a gente permite que entre mais de um familiar se for necessário. (MED-2)*

Como último tópico abordado como uma possibilidade de garantir o cuidado integral a essa população, os profissionais citaram meios de acesso ao serviço em que trabalham.

*Muitos acabam fazendo acompanhamento com a neuro, são crianças vinculadas ao atendimento ambulatorial especializado aqui no hospital que acabam em alguma outra urgência ou emergência procurando o nosso pronto-socorro, porque já são vinculadas ao serviço aqui. (ENF-2)*

*A gente tem as crianças que fazem acompanhamento com o neuropediatra e possuem o carimbo vermelho, então eles acabam procurando o atendimento aqui no PS Ped mesmo por outras demandas, não somente um ajuste medicamentoso, por exemplo. (ENF-3)*

No caso de crianças que foram encaminhadas ao serviço especializado e que mantêm acompanhamento após nascimento prematuro, estas possuem um vínculo com o hospital a partir do que é chamado de “Carimbo vermelho”. Sabendo que o Pronto-Socorro do Hospital Universitário não é de portas abertas para demandas espontâneas, o carimbo vermelho garante o acesso desse grupo de crianças ao PS Ped.

## DISCUSSÃO

Os achados desta pesquisa, ao evidenciar o papel da família, limitações e oportunidades na atenção às crianças com TEA em um Pronto-Socorro Pediátrico, dialogam com a complexidade da integralidade no contexto da urgência. A categorização dos dados permitiu uma análise crítica das condições estruturais, relacionais e organizacionais que podem dificultar a efetivação de um cuidado integral e humanizado.

A participação ativa da família no cuidado foi apontada pelos entrevistados como um elemento facilitador para a assistência à criança com TEA. Estratégias como o auxílio na abordagem da criança durante a aferição dos sinais vitais e a manutenção do diálogo entre profissionais e familiares foram citados como fundamentais para o êxito do atendimento. Tais práticas refletem os princípios do cuidado centrado na família, modelo que tem se mostrado eficaz na construção de vínculos e na redução do estresse dos cuidadores^(18)^.

Entretanto, lacunas na operacionalização do modelo de atenção à saúde centrado na família foram evidenciadas como uma entre as diversas limitações significativas relacionadas à assistência às crianças com TEA em Pronto-Socorro Pediátrico. Segundo os participantes de um estudo, existe dificuldade em encontrar algum momento favorável para prestar orientações quanto aos fluxos da rede de atenção e cuidados específicos, por exemplo. Essa falha da equipe em fornecer informações aos familiares pode intensificar o estresse dos cuidadores, situação que, por sua vez, tem repercussões diretas sobre o bem-estar e o comportamento da criança, impactando todo o processo de cuidado^(19)^.

A PNAISC reforça a necessidade de vínculos entre profissionais, criança e família como condição para a oferta de um cuidado integral e resolutivo^(4)^. Entretanto, os profissionais relatam que o tempo reduzido de permanência das crianças no serviço constitui um entrave importante para a construção desses vínculos. A literatura corrobora esse achado ao evidenciar que a lógica centrada na resolução imediata das demandas pode limitar o estabelecimento de relações terapêuticas mais profundas e significativas^(20)^.

Outra limitação citada pelos profissionais foi a inadequação da infraestrutura do serviço, evidenciada por ambientes ruidosos, iluminação intensa e compartilhamento de espaço com vários pacientes – fatores que se contrapõem às necessidades sensoriais de crianças com TEA. Essa realidade é observada em estudos internacionais que apontam o ambiente hospitalar como um espaço potencialmente desorganizador para essas crianças, intensificando quadros de agitação, resistência a procedimentos e risco de intervenções coercitivas, como a contenção física ou farmacológica^(10,11)^.

Estudo de revisão realizado em serviço de emergência pediátrica mostra que a contenção é justificável apenas em situações extremas e que seu uso frequente pode refletir falhas na adoção de abordagens preventivas e na capacitação das equipes de saúde. O manejo adequado exige conhecimento específico sobre a condição clínica da criança e estratégias não farmacológicas de regulação emocional, que muitas vezes estão ausentes na prática cotidiana das equipes^(21)^.

A análise das particularidades do cenário do estudo abordado pelos participantes do estudo revela a complexidade do atendimento às crianças com TEA no serviço de PS Ped, especialmente quando relacionado com a carência de leitos individuais e ambientes reservados para atendimento. Estudo qualitativo realizado com pais de crianças com TEA e profissionais do serviço de emergência aponta a inadequação dos espaços físicos como uma barreira significativa para um cuidado efetivo, especialmente relacionado a pacientes com demandas neuropsicológicas específicas, como é o caso de crianças com autismo^(9)^.

Além disso, a escassez de recursos humanos e a ausência de formação especializada sobre o transtorno foram amplamente apontadas pelos entrevistados como dificultadores da assistência integral e motivadores de sentimentos de preocupação, medo e insegurança. Uma pesquisa desenvolvida no mesmo cenário deste estudo também evidenciou dificuldades no cuidado relacionadas à infraestrutura do ambiente e ao reduzido número de profissionais da equipe de enfermagem por turno^(22)^. Pais e cuidadores frequentemente apontam a ausência de estratégias comunicativas adaptadas como um fator que aumenta o estresse da experiência em um Pronto-Socorro^(9)^.

As dificuldades relatadas pelos profissionais na linha de cuidado, como a demora no encaminhamento para serviços especializados e o início tardio do acompanhamento, revelam um cenário de fragilidade no acesso e na integralidade da atenção às crianças com TEA. Tais entraves comprometem não apenas o diagnóstico precoce e a intervenção oportuna, mas também a promoção da saúde e a reabilitação, pilares da atenção integral preconizada pela PNAISC^(7,23)^.

É importante destacar que muitas das fragilidades identificadas neste estudo extrapolam a atuação individual dos profissionais de saúde e estão relacionadas às responsabilidades da gestão em diferentes níveis do SUS^(14)^. No âmbito local, a gestão do Pronto-Socorro tem papel na adequação da infraestrutura, na definição de protocolos assistenciais e na promoção de capacitação continuada das equipes. Em uma perspectiva mais ampla, gestores municipais, estaduais e federais devem garantir investimentos estruturais e a implementação de políticas públicas que assegurem condições adequadas de trabalho e fluxos bem definidos de referência.

Assim, a superação das barreiras encontradas depende de decisões institucionais que integrem planejamento, financiamento e monitoramento, reforçando a corresponsabilidade entre profissionais e gestores ne efetivação da integralidade da atenção, conforme preconizado pela Política Nacional de Atenção às Urgências^(14)^.

Por fim, salienta-se que o trabalho em rede perpassa construções como o trabalho em equipe e a colaboração interprofissional, a fim de ofertar um projeto singular de cuidado complementado por todas as áreas multidisciplinares. Porém, o relato dos profissionais de saúde participantes do estudo expõe que, em alguns casos, esse trabalho em equipe multiprofissional não acontece, ficando algumas informações restritas aos profissionais médicos. Tais práticas devem ser evitadas, tendo em vista que apenas uma equipe multiprofissional completa possui o potencial de prestar todo o suporte necessário, singular e complementar às particularidades da manifestação do transtorno em cada criança^(24)^.

Em se tratando de estratégias adotadas pelos profissionais para o manejo de crianças com TEA no ambiente do Pronto-Socorro Pediátrico, o uso de ferramentas como as histórias sociais – histórias curtas e individualizadas que explicam uma situação social e representam tecnologia leve de cuidado – pelos profissionais de saúde pode ajudar pessoas com TEA na interpretação e na compreensão de situações sociais, como a internação hospitalar ou realização de algum procedimento. Esse tipo de estratégia pode melhorar a comunicação da equipe com a criança que não possui deficiência intelectual, auxiliando na melhor compreensão e redução do estresse que se relaciona com um ambiente hiper estimulante, como no caso de um Pronto-Socorro^(25)^.

Outro método utilizado pelos profissionais para melhorar o atendimento desse público é a flexibilização das rotinas e utilização do leito de isolamento para preservar a criança dos estímulos do serviço. Em um estudo de revisão retrospectivo, os profissionais entrevistados relataram a adoção de estratégias compensatórias, como as citadas pelos profissionais deste estudo para amenizar os efeitos dos estímulos excessivos e proporcionar um ambiente minimamente acolhedor. A criação de um ambiente mais tranquilo para atender as necessidades das crianças com TEA mostrou-se eficaz para auxiliar os profissionais a ofertar cuidados sensoriais^(26)^.

Além de planejamentos locais, o cuidado integral à criança com TEA requer articulação entre os diferentes pontos da RAS, da Atenção Primária à alta complexidade. A Rede de Atenção às Urgências, por exemplo, é fundamental e deve operar de forma interligada com os demais eixos estratégicos da PNAISC, inclusive aquele voltado às crianças com deficiência. Essa articulação visa garantir um cuidado contínuo e coordenado, capaz de responder às singularidades de cada criança e família^(7)^.

Instrumentos como o Cordão de Girassol e a CIPTEA contribuem para o reconhecimento das deficiências ocultas e o acesso prioritário aos serviços de saúde^(27,28)^, embora sua implementação e efetividade ainda careçam de maior uniformização no território nacional.

No âmbito da Atenção Primária, destaca-se o papel central dos profissionais médicos e enfermeiros na detecção precoce de sinais de alerta para o TEA, especialmente durante as consultas de puericultura. A utilização de instrumentos como o M-CHAT-R/F, recomendado pela Sociedade Brasileira de Pediatria e presente na Caderneta da Criança, possibilita uma triagem inicial de comportamentos sugestivos do transtorno em crianças de 16 a 30 meses^(29)^. Essa estratégia está em consonância com a Lei nº 13.438/2017, que estabelece diretrizes para a avaliação de riscos ao desenvolvimento psíquico na infância^(30)^. Entretanto, a efetividade dessa vigilância depende da formação dos profissionais, da continuidade do cuidado e da articulação com os demais níveis da rede.

Em relação ao acesso ao serviço terciário em questão, os profissionais citaram a adoção da instituição do “Carimbo Vermelho”, um instrumento disposto na Caderneta da Criança de pessoas que fazem acompanhamento com especialidades, como a neuropediatria, ou que nasceram prematuramente neste hospital. Essa estratégia configura-se como um meio de garantir o acesso de maior complexidade às crianças em um serviço que não trabalha com portas-abertas para demandas espontâneas e, sendo assim, vai ao encontro do preconizado pela integralidade e universalidade dentro do SUS^(6)^.

Assim, ao reconhecer os desafios e as possibilidades envolvendo a assistência às crianças com Transtorno do Espectro Autista em um Pronto-Socorro Pediátrico, este estudo reforça a necessidade de redes de atenção integradas e operantes, bem como de profissionais qualificados em todos os níveis de saúde. Ressalta-se que a qualificação da força de trabalho e a articulação entre os pontos da rede são condições indispensáveis para garantir o cuidado centrado na criança e sua família, conforme preconizado pelas diretrizes do SUS.

Em relação às limitações do estudo, a duração média das entrevistas, de cerca de 10 minutos, pode ser justificada pelo contexto do Pronto-Socorro Pediátrico, ambiente caracterizado por intensa demanda assistencial e pela limitação de tempo dos profissionais. Apesar disso, o rigor metodológico seguido pela pesquisadora e a pergunta disparadora direcionada possibilitaram a obtenção de relatos objetivos e centrados no fenômeno investigado.

Por fim, reconhece-se que o roteiro foi testado apenas com enfermeiros e técnicos de enfermagem do grupo de pesquisa das autoras, não contemplando os médicos. A avaliação da pergunta norteadora foi considerada pertinente devido à abrangência do olhar da Enfermagem na assistência em Pronto-Socorro. Além disso, durante a coleta de dados não foram relatadas dificuldades de compressão da pergunta pelos demais participantes, o que reforça a adequação do roteiro para todos os perfis profissionais incluídos.

## CONCLUSÃO

Concluiu-se que os profissionais de saúde do Pronto-Socorro Pediátrico reconhecem tanto potencialidades quanto fragilidades no cuidado às crianças com TEA. Entre os principais achados, destaca-se a percepção sobre o impacto da hospitalização nas famílias, frequentemente marcadas por estresse e dúvidas, o que reforça a importância de incluir os responsáveis no processo de cuidado como mediadores, realizando, ao mesmo tempo, educação em saúde.

Como a análise foi baseada na perspectiva dos profissionais, não é possível afirmar se o cuidado prestado é integral ou não, mas evidências apontam para fragilidades, como a ausência de protocolos padronizados, dificuldades na articulação com a RAS, limitações estruturais do serviço – especialmente a falta de ambientes com menor estímulo sensorial – e o dimensionamento insuficiente da equipe de enfermagem. Também foram relatadas inseguranças dos profissionais no manejo clínico, sinalizando a necessidade de capacitação específica e da educação permanente em saúde, estratégia essencial para ampliar competências técnicas e relacionais.

Por outro lado, emergiram estratégias adaptativas, como a flexibilização das regras institucionais, o uso do “Carimbo Vermelho” para garantir acesso ao serviço e a colaboração dos familiares, apontando caminhos possíveis para qualificar a assistência. Nesse sentido, destaca-se a relevância da realização de reuniões clínicas multiprofissionais como espaço de diálogo e troca de saberes, o que fortalece a tomada de decisão compartilhada. Ressalta-se, ainda, a necessidade de práticas de cuidado menos prescritivas e mais centradas na pessoa, nas quais a singularidade da criança e da família seja reconhecida. Essa perspectiva, em consonância com o princípio da integralidade do SUS, contribui para a humanização do atendimento, a redução de experiências traumáticas e a efetivação de um cuidado mais acolhedor e resolutivo.

Este estudo destaca-se pela originalidade no contexto brasileiro, ao abordar o cuidado a crianças com TEA em Pronto-Socorro Pediátrico, contribuindo de forma relevante para a literatura científica sobre o tema e oferecendo subsídios para reflexões futuras e arcabouço teórico para a gestão utilizar na criação de protocolos e novos fluxos de serviço.

Por fim, as implicações para a prática deste estudo contribuem tanto para a enfermagem, que precisa se instrumentalizar e desenvolver estratégias de acolhimento qualificadas, quanto para a gestão dos serviços, que deve investir em condições estruturais, dimensionamento adequado e políticas de capacitação, garantindo assim, um cuidado pautado na integralidade.

## DISPONIBILIDADE DE DADOS

Todo o conjunto de dados que dá suporte aos resultados deste estudo foi publicado no próprio artigo.

# Translation: Asistencia a niños con Trastorno del Espectro Autista: percepciones de los profesionales de la salud en un Servicio de Urgencias Pediátricas

**DOI:** 10.1590/1980-220X-REEUSP-2025-0350es

**Authors:** Camila Lovato de Figueiredo, Neila Santini de Souza, Aline Cammarano Ribeiro, Fernanda Luisa Buboltz, Hellen Thamara Quoos Santos, Eliane Tatsch Neves

**Affiliations:** 1 Universidade Federal de Santa Maria, Departamento de Enfermagem, Santa Maria, RS, Brasil.

**Keywords:** Salud Infantil, Enfermería Pediátrica, Trastorno del Espectro Autista, Servicio de Urgencia en Hospital, Grupo de Atención al Paciente

## Abstract

**Objetivo:**

Comprender la atención brindada a niños con Trastorno del Espectro Autista (TEA) en un Servicio de Urgencias Pediátricas desde la perspectiva de los profesionales de la salud.

**Método:**

Investigación cualitativa fundamentada en el marco de la atención integral. Se realizaron entrevistas narrativas a 17 profesionales del personal de enfermería y médico del Servicio de Urgencias Pediátricas de marzo a mayo de 2024. Las entrevistas fueron sometidas a análisis temático inductivo. El proyecto fue aprobado por el Comité de Ética en Investigación.

**Resultados:**

El estudio destacó tres categorías: la percepción de los profesionales sobre las familias frente al estrés y la falta de conocimiento sobre el TEA; las debilidades en la atención, marcadas por la ausencia de protocolos, dificultades en la coordinación en las Redes de Atención a la Salud (RAS), limitaciones estructurales e insuficiente dotación de personal de enfermería; y las estrategias adoptadas, como la colaboración familiar, las adaptaciones ambientales y la flexibilidad en las reglas.

**Conclusión:**

El cuidado de niños con TEA presenta desafíos estructurales, organizacionales y educativos que comprometen la integralidad de la atención. Se sugiere realizar inversiones en educación continua, mejoras de infraestructura y prácticas de atención centradas en la persona, de acuerdo con los principios del Sistema Único de Salud (SUS).

## INTRODUCCIÓN

El trastorno del espectro autista (TEA) se caracteriza por síntomas centrales relacionados con la comunicación social, comportamientos restringidos y repetitivos, además de comorbilidades relacionadas, incluyendo condiciones sensoriales, problemas de alimentación y comportamientos desafiantes^(1)^.

Los Centros para el Control y la Prevención de Enfermedades (CDC), organismo gubernamental de los Estados Unidos, identificaron una prevalencia de un niño con autismo por cada 36 niños de 8 años^(2)^. En Brasil, el Censo Demográfico de 2022 identificó que 2,4 millones de personas declararon haber recibido un diagnóstico de TEA (1,2% de la población brasileña)^(3)^.

Ante el creciente reconocimiento del TEA, es esencial debatir la estructura de los servicios de salud dirigidos a esta población. Teniendo esto en cuenta, la Red de Atención a la Salud (RAS) se organiza en puntos de atención articulados, incluyendo redes temáticas dirigidas a las principales demandas de la población. La Red de Atención a las Personas con Discapacidad se configura como uno de los ejes y establece directrices para la atención prioritaria a las personas con discapacidad, incluidas las personas con TEA. Sin embargo, su implementación aún presenta desigualdades, lo que compromete la eficacia de la asistencia, especialmente en entornos de urgencia y emergencia^(4)^.

Otro eje de la RAS es la Red de Atención a Urgencias, que se organiza a partir de la relación entre diversos servicios de atención a la salud, como el Servicio de Atención Móvil de Urgencias (SAMU), las Unidades de Atención de Urgencias (UPA) y el Componente Hospitalario, donde se inserta el Servicio de Urgencias Pediátricas, campo empírico de este estudio, caracterizado como una Puerta Hospitalaria de Urgencias de la RAS^(5)^.

Las RAS se basan en la integralidad de la atención, presente en la Ley 8.080/90, que regula el Sistema Único de Salud (SUS) y es un principio fundamental que comprende un enfoque organizativo de la asistencia. Este prioriza la horizontalidad de las acciones en salud y valora un modelo centrado en la persona, en sus demandas individuales y en la promoción de la humanización de la atención^(6)^.

La Política Nacional de Atención Integral a la Salud de la Niñez (PNAISC en la sigla en portugués) también refuerza la importancia de la integralidad de la atención como eje estructurante para la promoción de la salud infantil. La política reconoce que la salud de la niñez está influenciada por múltiples factores interrelacionados, siendo fundamental que los servicios de salud consideren todas las dimensiones de la vida en sus prácticas asistenciales^(7)^.

Dicho esto, en agosto de 2023 se llevó a cabo una investigación bibliográfica en las bases de datos de la Biblioteca Nacional de Medicina (PubMed) y Web of Science (WoS), utilizando la estrategia de búsqueda [*“Autism” AND “Child” AND “Emergency medical services”*], para la planificación y fundamentación de la investigación. En la suma de los resultados de las fuentes de evidencia, se identificaron 139 estudios, de los cuales 36 estaban alineados con el tema de la investigación y eran, en su gran mayoría, internacionales.

A partir del análisis de la literatura encontrada desde la perspectiva de la integralidad propuesta por la Ley 8.080/90 y por la PNAISC, es posible reconocer que, aunque la integralidad de la atención es un principio fundamental para la salud infantil y la necesidad de preparación del equipo se evidencia en estudios nacionales e internacionales^(8,9)^, el entorno de urgencias y emergencias presenta retos que exigen un enfoque adaptado a las necesidades de los niños con TEA.

La presencia de estímulos sensoriales, como sonidos, luces, olores y toques, en los entornos de urgencias, puede ser aversiva para estos niños, dada su hipersensibilidad^(10)^, siendo necesario el manejo adecuado de los profesionales ante las crisis de trastorno psíquico causadas por estos innumerables estímulos^(11)^. También se ha informado de la falta de conocimiento de los profesionales sobre las especificidades del trastorno, incluidos los signos y síntomas, las estrategias de comunicación eficaces y las formas adecuadas de involucrar a los familiares en el proceso de atención^(12)^.

Aunque existen directrices para la atención a esta población, siguen existiendo lagunas significativas en la comprensión y la práctica de la atención en los servicios de urgencias. Por lo tanto, es fundamental investigar la percepción y el enfoque de los profesionales de la salud en relación con estas cuestiones en el contexto de las urgencias, con el fin de identificar estrategias que mejoren la asistencia y promuevan una atención más eficaz y sensible a las necesidades de los niños con TEA y sus familias.

En este contexto, destaca el papel central de la enfermería, ya que esta categoría profesional mantiene un contacto continuo con los pacientes en el entorno hospitalario. Los enfermeros se encuentran en una posición estratégica para identificar las barreras en la atención e implementar estrategias que favorezcan una atención integral, sensible a las demandas sensoriales, conductuales y comunicativas de los niños con TEA en contextos de emergencia^(13)^.

Ante lo expuesto, se planteó la siguiente pregunta: ¿cómo se atiende a los niños con autismo en un servicio de urgencias pediátricas desde el punto de vista de los profesionales de la salud que trabajan allí? El objetivo era conocer la atención que reciben los niños con trastorno del espectro autista atendidos en servicios de urgencias pediátricas desde la perspectiva de los profesionales de la salud.

Se espera que los conocimientos obtenidos a partir de esta investigación contribuyan a la cualificación de la atención y a la atención integral de los niños con autismo en los servicios de urgencias pediátricas.

## MÉTODO

### Tipo de Estudio

Investigación cualitativa^(14)^ basada en el marco de referencia de la atención integral del Sistema Único de Salud (SUS), recogido en la Ley 8.080/90^(6)^ y en la Política Nacional de Atención Integral de la Niñez (PNAISC)^(7)^. Para la elaboración de este manuscrito se siguieron las recomendaciones de los Criterios consolidados para la presentación de informes de investigación cualitativa (COREQ)^(15)^.

### Escenario del Estudio

El estudio se llevó a cabo en un servicio de urgencias pediátricas de un hospital universitario de tamaño medio y alta complejidad, situado en Rio Grande do Sul. La estructura del servicio de urgencias pediátricas cuenta con siete camas, una de ellas de aislamiento y seis destinadas a la Unidad de Cuidados Convencionales. Además, la unidad dispone de una sala de emergencias con una camilla.

Este servicio es la principal vía de acceso a la atención pediátrica de urgencia y emergencia de la región centro-oeste del estado, siendo referencia para 45 municipios. El servicio está compuesto por pediatras y personal de enfermería que prestan atención las 24 horas, además de médicos residentes y un equipo multidisciplinario, que no es exclusivo del sector, sino que también incluye fisioterapeutas, trabajadores sociales y nutricionistas, por ejemplo, que pueden ser asignados para atender las necesidades de los niños y sus familias.

Los niños atendidos por este servicio son aquellos derivados por el SAMU (Sistema de ambulancias de urgencia pre-hospitalaria) en caso de emergencia grave; por servicios especializados, como neuropediatría; y niños que mantienen un seguimiento después de un parto prematuro. Tras su ingreso en la urgencia pediátrica, el flujo de estos niños puede producirse con el alta tras el tratamiento y la estabilización de los síntomas, o con la hospitalización en el servicio y el posterior traslado a la Unidad de Hospitalización Pediátrica o a la Unidad de Terapia Intensiva Pediátrica, según la gravedad del caso.

Un estudio realizado en la misma sala de urgencias reveló que se atendieron 3830 casos entre 2019 y 2023^(8)^. Entre ellas se incluyen emergencias como traumatismos, quemaduras graves y alteraciones metabólicas graves, además de cuestiones ambulatorias relacionadas con diagnósticos gastrointestinales, neurológicos, hematológicos y oncológicos, malformaciones congénitas, cardiovasculares y genitourinarias, asistencia por causas externas, casos quirúrgicos y, principalmente, asistencia por exacerbaciones de diagnósticos respiratorios^(8)^.

### Participantes y Criterios de Selección

La población de la investigación se definió por conveniencia, siendo un tipo de muestreo no probabilístico en el que los participantes se seleccionan en función de la facilidad de acceso y su potencial para responder a la investigación. Se buscó entrevistar a los profesionales de salud fijos de la Sala de Urgencias Pediátricas, siendo estos médicos especialistas en pediatría, enfermeros asistenciales, independientemente de su especialización, y técnicos en enfermería. Se tomó en consideración que estos son los componentes obligatorios del equipo básico de una Sala de Emergencias Pediátricas, de acuerdo con la Política Nacional de Atención a Urgencias^(16)^.

El criterio de inclusión fue ser profesional de la salud de las categorías mencionadas, activo en el escenario del estudio. Se excluyó a los profesionales que se encontraban en cualquier tipo de licencia o vacaciones durante el período de recolección de datos. La investigación se dio a conocer en el escenario del estudio y se invitó a los profesionales a participar en la investigación de forma aleatoria durante sus turnos de trabajo.

### Recolección de Datos

El procedimiento de recolección de datos se llevó a cabo de forma presencial, en el periodo comprendido entre marzo y mayo de 2024. La investigadora ya estaba familiarizada con el campo de investigación debido a las clases prácticas y las prácticas de la licenciatura en Enfermería. Por lo tanto, se volvió a acercar al servicio, momento en el que se presentó la investigación a los profesionales y, tras invitarlos a participar en el estudio, se les aclararon los objetivos de la investigación. De los 20 profesionales que trabajan en urgencia pediátrica, 12 del equipo de enfermería (7 enfermeros y 6 técnicos de enfermería) y 7 del equipo médico, se entrevistó a 6 enfermeros, 5 técnicos de enfermería y 6 médicos.

La entrevista narrativa se realizó con un guion previamente elaborado y se llevó a cabo una prueba piloto con enfermeros y técnicos de enfermería del grupo de investigación que trabajan con niños y adolescentes. El guion contenía preguntas para caracterizar al entrevistado y la pregunta que daba inicio a la entrevista narrativa: «¿Cómo percibe la atención a los niños autistas en el servicio de urgencias pediátricas?».

Las notas de campo se tomaron después de la entrevista y se incorporaron al proceso interpretativo, complementando los relatos transcritos. Estos registros ayudaron a contextualizar las declaraciones e identificar elementos del entorno que influyeron en la experiencia de los participantes, lo que contribuyó a la solidez del análisis.

Las entrevistas fueron realizadas únicamente por la investigadora graduada en Enfermería con experiencia en el ámbito y en investigación.

La recolección de datos se llevó a cabo en una sala reservada del servicio por la mañana, por la tarde y por la noche, y se grabó en audio, con una duración media de 10 minutos.

La suficiencia de los datos se definió según el criterio de saturación teórica, entendido como el punto en el que la información obtenida en las entrevistas comenzó a presentar recurrencia y consistencia, sin añadir nuevos elementos relevantes para los objetivos del estudio. Este proceso se supervisó de forma concomitante con la Recolección y el análisis preliminar de los datos. Tras la 17.ª entrevista, se observó una repetición de los contenidos y una convergencia de las declaraciones entre los diferentes participantes y categorías profesionales, lo que indicó la saturación y, en consecuencia, la suficiencia del material empírico. No hubo abandonos tras la aceptación de participar en la entrevista ni rechazos.

### Análisis y Tratamiento de los Datos

Las entrevistas grabadas se transcribieron íntegramente, sin devolverlas a los participantes, y se sometieron al análisis de contenido Temático Inductivo Reflexivo^(17)^, que consta de seis fases: 1) Familiarización con los datos; 2) Generación de códigos iniciales; 3) Búsqueda de temas; 4) Revisión de los temas identificados; 5) Definición y denominación de temas; 6) Elaboración del informe.

Se organizaron tres categorías de análisis: La familia de los niños con TEA en la sala de urgencias pediátricas desde la perspectiva de los profesionales de la salud; Debilidades relacionadas con el cuidado de los niños con TEA en la sala de urgencias pediátricas desde la perspectiva de los profesionales de la salud; y Estrategias adoptadas por los profesionales de la salud en la atención sanitaria de los niños con TEA en la sala de urgencias pediátricas.

### Aspectos Éticos

El estudio siguió todas las directrices y prerrogativas legales establecidas por el Consejo Nacional de Salud y fue aprobado por el Comité de Ética en Investigación (CEP) de la institución, con el número de dictamen 6.512.409/2023. Todos los participantes recibieron explicaciones claras y objetivas sobre la naturaleza de la investigación, sus propósitos y las orientaciones para la realización de las entrevistas. Se entregó el Formulario de Consentimiento Libre e Informado (FCLI), que se firmó por duplicado, entregándose una copia al participante y conservando la otra la investigadora.

Con el fin de mantener el anonimato de los participantes, se utilizaron códigos referidos a la profesión de los profesionales, adoptando ENF para enfermero, TE para técnico de enfermería y MED para médico, además de una numeración en orden ascendente, según el orden en que se realizó la entrevista.

## RESULTADOS

A partir de la caracterización de los participantes, se evidenció que, de los 17 incluidos, seis eran enfermeros, cinco técnicos de enfermería y seis médicos, entre los cuales predominaba el sexo femenino, con 16 (94,12%) del total.

Los participantes tenían entre 27 y 56 años de edad, con una media de 42,30 años, y el tiempo de formación en sus respectivas profesiones variaba entre 4 y 29 años, con una media de 16,41 años. Por su parte, el tiempo de actuación en el Servicio de Urgencias Pediátricas variaba entre 1 mes y 23 años, con una media de 7,52 años.

En cuanto a la formación de posgrado o posgrado técnico, el 100% (17) de los participantes afirmaron tenerla, y en 8 de los casos las especializaciones eran en el área de actuación de estos profesionales, como Pediatría y Urgencias y Emergencias.

Los resultados obtenidos a partir del análisis de los datos obtenidos en las entrevistas se sintetizaron en 3 categorías temáticas que se describen a continuación.

*La familia de los niños con TEA en Urgencias Pediátricas desde la perspectiva de los profesionales de la salud*

Los profesionales relataron su percepción sobre las familias de los niños con autismo durante las hospitalizaciones en el servicio, el impacto de salir del entorno al que el niño está acostumbrado y la falta de conocimiento de las familias sobre el TEA.

*Muchas familias se estresan mucho con la dificultad de manejar al paciente, especialmente porque cada vez que sale de su contexto, se vuelve más difícil. Algunos procedimientos son un poco más difíciles de realizar para la familia. Esto, en consecuencia, retroalimenta el estrés del paciente. (MED-1)*

*A veces los padres no tienen mucho conocimiento o no tienen mucho interés, esto varía mucho. (TE-1)*

*La familia no siempre está preparada, a veces las madres llegan aquí con una sospecha de diagnóstico, no siempre tienen el diagnóstico formado y se conocen los cuidados diferenciados para estos niños. Entonces tienen dificultades, llegan aquí llenos de dudas y aquí en la sala de urgencias no es un lugar donde tengamos mucho tiempo para hablar de eso. (ENF-5)*

Los participantes mencionaron la inclusión del familiar en la atención durante la consulta en el servicio de urgencias pediátricas y su actuación como facilitador en este proceso.

*Pedimos mucha ayuda a los padres, porque no llegamos ya poniendo el termómetro, por ejemplo, cuando vamos a ver los signos. Se lo doy a la madre para que se lo ponga al niño. Entonces tratamos de supervisar más a la madre, para que no se pongan nerviosos y agitados. (TE-2)*

*Intentamos atender a los niños respetando sus limitaciones. A veces, si se trata de un paciente poco colaborador, pedimos ayuda a los padres para cogerlo en brazos y poder hacerle un examen físico. Intentamos adaptarnos para no generar más estrés al niño y a la familia. (MED-4)*

En resumen, los participantes observan, durante la atención a niños con TEA, el estrés y el desconocimiento de los cuidadores sobre el trastorno y perciben la importancia de involucrar a los responsables del cuidado, buscando escuchar del acompañante cuáles son los límites del niño.

*Debilidades relacionadas con el cuidado de niños con TEA en la sala de urgencias pediátricas desde la perspectiva de los profesionales de la salud*

En cuanto a las limitaciones presentes en el contexto de la sala de urgencias pediátricas, los profesionales mencionaron algunas barreras relacionadas con la dificultad de manejar a estos niños, como el estigma asociado a las inyecciones y las punciones venosas, además de la desorganización psíquica causada por el entorno hospitalario.

*Dependiendo de cómo se encuentren [los niños], si sienten dolor, malestar, entonces suelen mostrar más resistencia al contacto, sobre todo porque ya lo relacionan con la punción venosa. (ENF-1)*

*Llegan con miedo al equipo de salud, ya están nerviosos por las inyecciones. (ENF-3)*

*Están muy estresados por estar aquí, en el entorno hospitalario, más estresados que los demás. (TE-3)*

Los profesionales informaron de factores relacionados con el acceso a servicios especializados, el diagnóstico precoz y el trabajo en equipo como limitaciones que existen en el servicio y en la sociedad:

*Todo el fallo está en la red. Llegó una madre con un niño autista de forma espontánea porque la maestra le dijo que viniera aquí. La madre dijo que la maestra le había comentado que hay otros dos compañeros que son especiales, y que la maestra quería obtener un diagnóstico para poder solicitar un monitor. Hablamos con ella para que se pusiera en contacto con la unidad básica, el médico podría solicitarlo a un pediatra o directamente a un neurólogo. Pero la evaluación con el neurólogo está tardando un año y medio aquí y en el sector privado seis meses. Entonces, le dijimos que podía optar por hacerlo en el sector privado, pero que sería importante que mantuviera el vínculo aquí con el hospital, porque después es difícil entrar. Creo que hay muchos niños que lo necesitan y que no pueden acceder al servicio. (ENF-4)*

*Tenemos especialistas, pero tardan un poco y, a menudo, no hablan con el equipo de enfermería, hablan con el equipo médico y, a veces, ni siquiera sabemos qué hacer con estos niños. (ENF-6)*

La infraestructura de la sala de urgencias pediátricas también se menciona como un obstáculo, ya que el área de hospitalización no cuenta con un entorno privado más allá del aislamiento, lo que dificulta la protección de los niños con TEA frente a los innumerables estímulos inherentes a la unidad.

Además, citan la cantidad de personal, con un equipo de enfermería compuesto por un enfermero y un técnico de enfermería por turno para cinco camas y la atención a las demandas externas, como otro reto.

*Es difícil porque no tenemos consultorio, cuando está lleno no tenemos dónde atender. (MED-3)*

*No tenemos un espacio físico muy bueno que nos permita atender sin ruido, sin muchos estímulos, porque cuando tienen que estar en la sala hay muchas camas, lo que perturba a los niños autistas. (MED-6)*

*Solo somos dos aquí [una enfermera y una técnica de enfermería], por lo que en algunos procedimientos es muy difícil que podamos actuar. Si se trata de un niño autista más agresivo, tenemos dificultades [...] a veces incluso tenemos que pedir ayuda a otros compañeros de otros sectores. (TE-4)*

Por último, algunos profesionales mencionaron que se sienten preocupados, con dificultades y miedo al atender a un niño con TEA, lo que indica la necesidad de formación para acompañar a estos niños.

*Solo con los agresivos tenemos más dificultades, yo misma tengo miedo de que nos peguen, porque hay niños que son más grandes que yo. (TE-5)*

*Creo que el equipo en sí mismo atiende bien a los pacientes, quizá lo que podría mejorarse un poco es la formación que recibimos sobre cómo tratar a estos niños, porque yo misma, como profesional, nunca he recibido formación ni nada por el estilo. (ENF-5)*

Estas limitaciones dificultan la atención integral, ya que la falta de un entorno adecuado que satisfaga las necesidades específicas de cualquier población, así como los sentimientos de preocupación, miedo e inseguridad, además de la falta de cualificación específica, terminan dificultando la prestación de una atención cualificada e integral.

*Estrategias adoptadas por los profesionales de la salud en la atención médica de niños con TEA en la sala de urgencias pediátricas*

Algunos profesionales relataron sus percepciones sobre la actuación del equipo durante la atención a un niño con TEA.

*Aquí, en la sala de urgencias, percibo que el equipo está más acostumbrado a las particularidades de estos pacientes, por lo que creo que la atención, dentro de lo posible, es adecuada. (MED-1)*

*En general, creo que puedo manejarlo bien y el equipo también colabora mucho en este sentido. (ENF-3)*

Algunos participantes mencionaron que ellos mismos, o el equipo en general, no tienen problemas significativos relacionados con la atención de niños con TEA y utilizan algunas estrategias adoptadas para facilitar la atención.

*Tuve un caso de sospecha de apendicitis en una niña autista con un grado importante de dependencia y le administramos sedantes antes de enviarla a la tomografía, para evitar que se agitara con el cambio de entorno. (MED-1)*

*Cuando el niño es muy agresivo o tiene mucha hipersensibilidad, lo aislamos cuando es posible, o aquí en estas camas de consulta, para intentar que haya menos estímulos, con menos gente pasando. (MED-5)*

*Aquí en Urgencias tenemos algunas reglas: cuando atendemos a un niño, solo puede entrar un acompañante, porque tenemos pocas camas y el espacio físico es reducido, pero cuando tenemos un paciente con TEA permitimos que entre más de un familiar si es necesario. (MED-2)*

Como último tema abordado como una posibilidad de garantizar la atención integral a esta población, los profesionales mencionaron los medios de acceso al servicio en el que trabajan.

*Muchos terminan haciendo un seguimiento con el neurólogo, son niños vinculados a la atención ambulatoria especializada aquí en el hospital que terminan en alguna otra urgencia o emergencia buscando nuestro servicio de urgencias, porque ya están vinculados al servicio aquí. (ENF-2)*

*Tenemos niños que son atendidos por el neurólogo pediátrico y tienen el sello rojo, por lo que acaban acudiendo a la atención aquí en la sala de urgencias, incluso por otras demandas, no solo por un ajuste de la medicación, por ejemplo. (ENF-3)*

En el caso de los niños que han sido derivados al servicio especializado y que siguen en seguimiento tras un parto prematuro, estos tienen un vínculo con el hospital a partir de lo que se denomina «sello rojo». Sabiendo que la sala de urgencias del Hospital Universitario no está abierta a demandas espontáneas, el sello rojo garantiza el acceso de este grupo de niños a la sala de urgencias pediátricas.

## DISCUSIÓN

Los hallazgos de esta investigación, al evidenciar el papel de la familia, las limitaciones y las oportunidades en la atención a los niños con TEA en una sala de urgencias pediátricas, dialogan con la complejidad de la integralidad en el contexto de la urgencia. La categorización de los datos permitió un análisis crítico de las condiciones estructurales, relacionales y organizativas que pueden dificultar la efectividad de una atención integral y humanizada.

Los entrevistados señalaron que la participación activa de la familia en la atención era un elemento facilitador para la asistencia al niño con TEA. Se mencionaron estrategias como la ayuda en el acercamiento al niño durante la medición de los signos vitales y el mantenimiento del diálogo entre los profesionales y los familiares como fundamentales para el éxito de la atención. Estas prácticas reflejan los principios de la atención centrada en la familia, un modelo que ha demostrado su eficacia en la construcción de vínculos y en la reducción del estrés de los cuidadores^(18)^.

Sin embargo, se evidenciaron brechas en la puesta en práctica del modelo de atención sanitaria centrada en la familia como una de las diversas limitaciones significativas relacionadas con la atención a los niños con TEA en los servicios de urgencias pediátricas. Según los participantes en un estudio, existe la dificultad de encontrar un momento favorable para proporcionar orientación sobre los flujos de la red de atención y cuidados específicos, por ejemplo. Esta falta de información por parte del equipo a los familiares puede intensificar el estrés de los cuidadores, situación que, a su vez, tiene repercusiones directas en el bienestar y el comportamiento del niño, lo que afecta a todo el proceso de atención^(19)^.

La PNAISC refuerza la necesidad de vínculos entre los profesionales, el niño y la familia como condición para ofrecer una atención integral y resolutiva^(4)^. Sin embargo, los profesionales informan que el reducido tiempo de permanencia de los niños en el servicio constituye un obstáculo importante para la construcción de estos vínculos. La literatura corrobora este hallazgo al evidenciar que la lógica centrada en la resolución inmediata de las demandas puede limitar el establecimiento de relaciones terapéuticas más profundas y significativas^(20)^.

Otra limitación citada por los profesionales fue la inadecuación de la infraestructura del servicio, evidenciada por entornos ruidosos, iluminación intensa y el uso compartido del espacio con varios pacientes, factores que se oponen a las necesidades sensoriales de los niños con TEA. Esta realidad se observa en estudios internacionales que señalan el entorno hospitalario como un espacio potencialmente desorganizador para estos niños, lo que intensifica los cuadros de agitación, la resistencia a los procedimientos y el riesgo de intervenciones coercitivas, como la contención física o farmacológica^(10,11)^.

Un estudio de revisión realizado en un servicio de urgencias pediátricas muestra que la contención solo se justifica en situaciones extremas y que su uso frecuente puede reflejar fallos en la adopción de enfoques preventivos y en la capacitación de los equipos de salud. El manejo adecuado requiere conocimientos específicos sobre la condición clínica del niño y estrategias no farmacológicas de regulación emocional, que a menudo están ausentes en la práctica cotidiana de los equipos^(21)^.

El análisis de las particularidades del escenario del estudio abordado por los participantes revela la complejidad de la atención a los niños con TEA en el servicio de urgencias pediátricas, especialmente cuando se relaciona con la falta de camas individuales y entornos reservados para la atención. Un estudio cualitativo realizado con padres de niños con TEA y profesionales del servicio de urgencias señala la inadecuación de los espacios físicos como una barrera significativa para una atención eficaz, especialmente en relación con pacientes con demandas neuropsicológicas específicas, como es el caso de los niños con autismo^(9)^.

Además, la escasez de recursos humanos y la falta de formación especializada sobre el trastorno fueron ampliamente señaladas por los entrevistados como obstáculos para la atención integral y motivadores de sentimientos de preocupación, miedo e inseguridad. Una investigación desarrollada en el mismo escenario de este estudio también evidenció dificultades en la atención relacionadas con la infraestructura del entorno y el reducido número de profesionales del equipo de enfermería por turno^(22)^. Los padres y cuidadores suelen señalar la falta de estrategias comunicativas adaptadas como un factor que aumenta el estrés de la experiencia en una sala de urgencias^(9)^.

Las dificultades relatadas por los profesionales de la atención sanitaria, como la demora en la derivación a servicios especializados y el inicio tardío del seguimiento, revelan un panorama de fragilidad en el acceso y la integralidad de la atención a los niños con TEA. Estos obstáculos comprometen no solo el diagnóstico precoz y la intervención oportuna, sino también la promoción de la salud y la rehabilitación, pilares de la atención integral preconizada por el PNAISC^(7,23)^.

Es importante destacar que muchas de las fragilidades identificadas en este estudio trascienden la actuación individual de los profesionales de la salud y están relacionadas con las responsabilidades de la gestión en diferentes niveles del SUS^(14)^. A nivel local, la gestión de los servicios de urgencias desempeña un papel importante en la adecuación de la infraestructura, la definición de protocolos de atención y la promoción de la formación continua de los equipos. Desde una perspectiva más amplia, los gestores municipales, estatales y federales deben garantizar inversiones estructurales y la implementación de políticas públicas que aseguren condiciones de trabajo adecuadas y flujos de referencia bien definidos.

Así, la superación de las barreras encontradas depende de decisiones institucionales que integren la planificación, la financiación y el seguimiento, reforzando la corresponsabilidad entre profesionales y gestores en la efectividad de la integralidad de la atención, tal y como recomienda la Política Nacional de Atención a Urgencias^(14)^.

Por último, cabe destacar que el trabajo en red abarca conceptos como el trabajo en equipo y la colaboración interprofesional, con el fin de ofrecer un proyecto de atención único complementado por todas las áreas multidisciplinarias. Sin embargo, el relato de los profesionales de la salud que participaron en el estudio revela que, en algunos casos, este trabajo en equipo multiprofesional no se lleva a cabo, quedando cierta información restringida a los profesionales médicos. Estas prácticas deben evitarse, ya que solo un equipo multiprofesional completo tiene el potencial de proporcionar todo el apoyo necesario, singular y complementario a las particularidades de la manifestación del trastorno en cada niño^(24)^.

En lo que respecta a las estrategias adoptadas por los profesionales para el manejo de niños con TEA en el entorno de la sala de urgencias pediátricas, el uso de herramientas como las historias sociales —historias cortas e individualizadas que explican una situación social y representan una tecnología de cuidado ligera— por parte de los profesionales de la salud puede ayudar a las personas con TEA a interpretar y comprender situaciones sociales, como la hospitalización o la realización de algún procedimiento. Este tipo de estrategia puede mejorar la comunicación del equipo con el niño que no tiene discapacidad intelectual, ayudando a comprender mejor y reducir el estrés relacionado con un entorno hiperestimulante, como en el caso de una sala de urgencias^(25)^.

Otro método utilizado por los profesionales para mejorar la atención a este público es la flexibilización de las rutinas y el uso de camas de aislamiento para proteger al niño de los estímulos del servicio. En un estudio de revisión retrospectivo, los profesionales entrevistados informaron de la adopción de estrategias compensatorias, como las citadas por los profesionales de este estudio, para mitigar los efectos de los estímulos excesivos y proporcionar un entorno mínimamente acogedor. La creación de un entorno más tranquilo para atender las necesidades de los niños con TEA resultó eficaz para ayudar a los profesionales a ofrecer cuidados sensoriales^(26)^.

Además de la planificación local, la atención integral al niño con TEA requiere la articulación entre los diferentes puntos de la RAS, desde la atención primaria hasta la alta complejidad. La Red de Atención a Urgencias, por ejemplo, es fundamental y debe operar de manera interconectada con los demás ejes estratégicos de la PNAISC, incluido el dirigido a los niños con discapacidad. Esta articulación tiene como objetivo garantizar una atención continua y coordinada, capaz de responder a las singularidades de cada niño y familia^(7)^.

Instrumentos como el Cordón de Girasol y la Cédula de identificación de personas con trastorno del espectro autista (CIPTEA) contribuyen al reconocimiento de las discapacidades ocultas y al acceso prioritario a los servicios de salud^(27,28)^, aunque su implementación y eficacia aún necesitan una mayor uniformidad en el territorio nacional.

En el ámbito de la atención primaria, cabe destacar el papel central de los médicos y enfermeros en la detección precoz de señales de alerta del TEA, especialmente durante las consultas de puericultura. El uso de instrumentos como el M-CHAT-R/F, recomendado por la Sociedad Brasileña de Pediatría y presente en el Cuaderno de la Niñez, permite una detección inicial de comportamientos sugestivos del trastorno en niños de 16 a 30 meses^(29)^. Esta estrategia está en consonancia con la Ley n.º 13.438/2017, que establece directrices para la evaluación de los riesgos para el desarrollo psíquico en la infancia^(30)^. Sin embargo, la eficacia de esta vigilancia depende de la formación de los profesionales, la continuidad de la atención y la articulación con los demás niveles de la red.

En relación con el acceso al servicio terciario en cuestión, los profesionales mencionaron la adopción de la institución del «Sello Rojo», un instrumento dispuesto en la Cartilla del Niño de personas que reciben seguimiento con especialidades, como la neuropediatría, o que nacieron prematuramente en este hospital. Esta estrategia se configura como un medio para garantizar un acceso de mayor complejidad a los niños en un servicio que no trabaja con puertas abiertas para demandas espontáneas y, por lo tanto, va en encuentro con lo recomendado por la integralidad y la universalidad dentro del SUS^(6)^.

Así, al reconocer los retos y las posibilidades que implica la atención a los niños con trastorno del espectro autista en un servicio de urgencias pediátricas, este estudio refuerza la necesidad de redes de atención integradas y operativas, así como de profesionales cualificados en todos los niveles de la salud. Cabe destacar que la cualificación de la fuerza de trabajo y la articulación entre los puntos de la red son condiciones indispensables para garantizar una atención centrada en el niño y su familia, tal y como recomiendan las directrices del SUS.

En cuanto a las limitaciones del estudio, la duración media de las entrevistas, de unos 10 minutos, puede justificarse por el contexto de la sala de urgencias pediátricas, un entorno caracterizado por una intensa demanda asistencial y por la limitación de tiempo de los profesionales. A pesar de ello, el rigor metodológico seguido por la investigadora y la pregunta desencadenante dirigida permitieron obtener relatos objetivos y centrados en el fenómeno investigado.

Por último, se reconoce que el guion solo se probó con enfermeros y técnicos de enfermería del grupo de investigación de las autoras, sin incluir a los médicos. La evaluación de la pregunta orientadora se consideró pertinente debido al alcance de la perspectiva de la enfermería en la atención en urgencias. Además, durante la recopilación de datos no se informaron dificultades para comprender la pregunta por parte de los demás participantes, lo que refuerza la idoneidad del guion para todos los perfiles profesionales incluidos.

## CONCLUSIÓN

Se concluyó que los profesionales de salud de la sala de urgencias pediátricas reconocen tanto las potencialidades como las debilidades en la atención a los niños con TEA. Entre los principales hallazgos, destaca la percepción sobre el impacto de la hospitalización en las familias, a menudo marcadas por el estrés y las dudas, lo que refuerza la importancia de incluir a los responsables en el proceso de atención como mediadores, realizando al mismo tiempo educación en salud.

Dado que el análisis se basó en la perspectiva de los profesionales, no es posible afirmar si la atención prestada es integral o no, pero las evidencias apuntan a debilidades, como la ausencia de protocolos estandarizados, las dificultades en la articulación con la RAS, las limitaciones estructurales del servicio —especialmente la falta de entornos con menor estímulo sensorial— y el tamaño insuficiente del equipo de enfermería. También se han señalado inseguridades de los profesionales en el manejo clínico, lo que indica la necesidad de una capacitación específica y una educación permanente en salud, estrategia esencial para ampliar las competencias técnicas y relacionales.

Por otro lado, han surgido estrategias adaptativas, como la flexibilización de las normas institucionales, el uso del «Sello Rojo» para garantizar el acceso al servicio y la colaboración de los familiares, lo que apunta a posibles vías para mejorar la calidad de la asistencia. En este sentido, cabe destacar la importancia de celebrar reuniones clínicas multiprofesionales como espacio de diálogo e intercambio de conocimientos, lo que fortalece la toma de decisiones compartida. Cabe destacar, además, la necesidad de prácticas de atención menos prescriptivas y más centradas en la persona, en las que se reconozca la singularidad del niño y de la familia. Esta perspectiva, en consonancia con el principio de integralidad del SUS, contribuye a la humanización de la atención, a la reducción de experiencias traumáticas y a la efectividad de una atención más acogedora y resolutiva.

Este estudio se destaca por su originalidad en el contexto brasileño, al abordar la atención a niños con TEA en urgencias pediátricas, contribuyendo de manera relevante a la literatura científica sobre el tema y ofreciendo bases para futuras reflexiones y un marco teórico para que la dirección lo utilice en la creación de protocolos y nuevos flujos de servicio.

Por último, las implicaciones prácticas de este estudio contribuyen tanto a la enfermería, que necesita dotarse de herramientas y desarrollar estrategias de acogida cualificadas, como a la gestión de los servicios, que debe invertir en condiciones estructurales, dimensionamiento adecuado y políticas de capacitación, garantizando así una atención basada en la integralidad.

## DISPONIBILIDAD DE DATOS

Todo el conjunto de datos que apoya los resultados de este estudio fue publicado en el propio artículo.
